# Supplementary material for: Political expression of academics on Twitter
Source: Nat Hum Behav. 2025 Jun 3;9(9):1815–32. doi: 10.1038/s41562-025-02199-1 (PMC12454119; doi:10.1038/s41562-025-02199-1)
Supplement: Supplementary file 1 — Supplementary Notes 1–5, Tables 1–14 and Figs. 1–23. [file 41562_2025_2199_MOESM1_ESM.pdf]

nature human behaviour

Supplementary information

https://doi.org/10.1038/s41562-025-02199-1

Political expression of academics on Twitter

In the format provided by the authors and unedited

Contents

1 Validation of stance detection **3**

2 Validation of topic detection **7**

3 Validation of gender classification **8**

4 Comparison with opinion poll **9**

5 Additional Results **11**

5.1 Granular Field Level Distribution 11

5.2 Correlates of Egocentric Expression 12

5.3 Robustness of Subgroup Differences in Political Stances After Controlling for Tone and Style Variables 13

5.4 Robust Trends - Partialling Out Individual Fixed Effects 15

6 Tables **17**

7 Figures **27**

List of Tables

1 Power-Law Fit Parameters for Academic Influence Metrics 17

2 Normality and Variance Homogeneity Checks for Climate Action and Climate Narrative Comparisons 18

3 Normality and Variance Homogeneity Checks for Cultural Liberalism and Economic Collectivism Comparisons 19

4 Normality and Variance Homogeneity Checks for Behavioral Feature Comparisons 20
5 Normality and Variance Homogeneity Checks for Temporal Dynamics in Academic Expression . . . 21
6 Normality and Variance Homogeneity Checks for Comparisons of Academic vs. Non-Academic Users . . . 22
7 Raw Correlations Between Stances and Emotionality, Egocentrism, and Toxicity . 23
8 Tweet level Summary Statistics . . . 24
9 Balancedness Test: Comparison of Balanced and Unbalanced Populations 25
11 Evaluation Metrics for Stance Detection . . . 26
12 Comparison of Agreement and F1 Scores Across GPT Models . . . 26
10 Random Sample of Terms for Topics Detected using GPT-4 . . . 48
13 Evaluation Metrics for Topic Detection Validation . . . 49
14 Evaluation Metrics for Gender Classification . . . 49

List of Figures

1 Distribution of **Egocentric** Expressions by field . . . 27
2 Distribution of **Climate Action** Expression by field . . . 28
3 Distribution of **Behavioural Adjustment** Narrative in Climate Action by field . . 29
4 Distribution of **Techno-Optimism** Narrative in Climate Action by field . . . 30
5 Distribution of **Cultural Liberalism** Expressions by field . . . 31
6 Distribution of **Economic Collectivism** Expressions by field . . . 32
7 Correlates of Egocentric Expression . . . 33
8 Correlates of Political Expression . . . 34

9 Group Comparisons of Climate-Related Stances Controlling for Different Expressions . . . 35
10 Group Comparisons of Economic and Cultural Stances Controlling for Different Expressions . . . 36
11 Academic Expression Over Time by Gender . . . 37
12 Academic Expression Over Time by Fields . . . 38
13 Academic Expression Over Time by Country (US vs. Non-US) . . . 39
14 Academic Expression Over Time by Twitter Reach vs. Academic Credibility . . . 40
15 Average Number of Topical Tweets Over Time (2016–2022) . . . 41
16 Total Counts of Topical Tweets Over Time (2016–2022) . . . 42
17 Trends in Subcomponents of Cultural Liberalism and Economic Collectivism . . . 43
18 Within-User Trends in Academic Expression Over Time (Main Sample) . . . 44
19 Within-User Trends in Academic Expression by Gender . . . 45
20 Within-User Trends in Academic Expression by Field of Study . . . 46
21 Within-User Trends in Academic Expression by Country of Institution . . . 47
22 Spatio-temporal correlation between Twitter and GSS stance on Climate Action . . . 50
23 Spatio-temporal correlation between Twitter and GSS stance on Abortion Rights 50

1 Validation of stance detection

This validation study employs the dataset from the ACL SemEval-2016 Task 6 - Stance Detection in Tweets.[1] This dataset provides an ideal ground for testing the accuracy and reliability of our stance detection model developed using GPT-based large language models. SemEval-2016 Task 6 comprises two distinct tasks: Task A (Supervised Stance Detection): This task involves

predicting stances (pro, anti, and neutral) towards various targets, including the Feminist Movement, Hillary Clinton, and Legalization of Abortion. The dataset consists of 40,317 hand-coded labels from 137 humans. Participants in the challenge were provided with 70% of these labels for training purposes, with the remaining 30% reserved for testing. Task B (Approximate Zero-Shot Labelling): In this task, participants were given around 72,000 unlabelled tweets on the topic *Donald Trump* to predict the stance without explicit training on this specific target. The primary evaluation metric used in SemEval-2016 Task 6 is the average F1 score per target, calculated as follows:

$$F_{avg} = (F_{pro} + F_{anti})/2 \tag{1}$$

where $F_{pro} = \frac{2 \times P_{proi} \times R_{pro}}{P_{pro} \times R_{pro}}$, $F_{anti} = \frac{2 \times P_{anti} \times R_{anti}}{P_{anti} \times R_{anti}}$, P is precision and R is recall. For validating our stance detection method described in section 3.2, we applied the GPT-based model to categorize the stance of tweets in both tasks. The model's predictions were then compared to the human labels in the dataset. The results in Supplementary Table 11 demonstrate that our GPT-based stance detection model achieves F_{avg} score of 84 to 92. This indicates a high level of reliability and accuracy, aligning closely with human judgments. These findings provide a strong validation of our approach, reinforcing its capability for accurate and nuanced stance detection in tweets. The model performance, particularly in the zero-shot labelling task, underscores its potential for generalization and application in diverse contexts beyond the initial training set.

In comparison to traditional stance detection models in SemEval-2016 Task 6, our GPT-3.5 Turbo-based approach shows marked improvements. The best model, a Recurrent Neural Network (RNN) with transfer learning, achieved a F_{avg} score of 67.82% on the Feminist Movement target,[1,2] while others, like a genetic algorithm ensemble for Hillary Clinton, reached 66.83%,

and character-word CNNs managed 63.32% on Legalization of Abortion. In Task B, MITRE's rule-based CNN for Donald Trump performed at 56.28%.[2] These older models required extensive feature engineering and static lexicons, limiting adaptability to new contexts. By contrast, our zero-shot GPT approach leverages the flexibility of large language models, eliminating manual tuning and capturing nuanced, evolving stances across targets. Our F_{avg} scores range from 79.52% to 92.44%, significantly outperforming traditional methods while maintaining high precision and recall without the need for extensive feature design, highlighting. the robustness of LLMs in dynamic environments like Twitter.

Additionally, we compare with GPT-4 (a model trained on larger parameters). We show that GPT-4 performs similarly to GPT-3.5, since the tasks are fairly straightforward. GPT-4 outperforms GPT-3.5 only on one of the four topics, i.e., on Legalization of Abortion, with an F_{avg} score of 84.36, compared to 79.52 from GPT-3.5 Turbo. Since the performance across the four topics is not that different, we are confident that GPT-3.5 Turbo is sufficient for the labelling task.

Comparison Across Multiple Models and Iterations In addition to the validation using the SemEval-2016 Task 6 dataset, we further evaluated the reliability and consistency of stance detection by comparing outputs across multiple GPT-based models. Specifically, we analyzed the agreement and performance of four models: GPT-3.5-turbo, GPT-4, GPT-4o, and GPT-4o-mini.

We first sampled 400 random tweets per topic per stance from authors in our main analysis sample, resulting in a dataset of 8,400 unique tweets (400 tweets times seven topics times three stance categories). Each tweet was labeled over 10 iterations by all four models, leading to a total of 336,000 predictions. After filtering to retain only the tweets where all iterations across the four models resulted in a valid stance (pro, anti, or neutral), we were left with 75.25% of the

tweets, or 63,210 predictions per model. The exclusion of tweets involved cases where at least one iteration of a model labeled the tweet as unrelated, ensuring that we compared stances for tweets that almost certainly belonged to a specific topic.

Within this corpus, we conducted two types of evaluations: the measurement of agreement rate and the measurement of the average F1 score (F_{avg}) as defined in equation 1. When calculating F_{avg}, we treated one model's predictions as the "true" set of labels and compared the other models against it. This approach allows us to assess how well each model performs relative to a chosen reference model in terms of precision and recall, providing insight into the accuracy of stance classification. The agreement rate, on the other hand, measures the consistency between models by calculating the proportion of identical stance predictions across different models. While F_{avg} focuses on how closely each model aligns with a reference standard, the agreement rate highlights the degree of consensus among the models. A high agreement rate indicates that the models are generally consistent in their predictions, while a high F_{avg} score suggests that a model performs well relative to the reference model in correctly classifying stances.

We conducted these evaluations for two types of stance data. The first approach treated each iteration for each tweet as a unique instance, providing an overarching view of model performance across different runs. The second approach calculated the modal stance across the 10 iterations for each model, offering a consolidated view of each model's typical output.

The results show strong consistency across models, with GPT-4o, the most recent model, yielding the highest agreement rates and F_{avg} scores. Notably, both metrics increase as the quality of the compared model improves, from GPT-4o-mini to GPT-4 to GPT-4o. This pattern aligns with expectations given the superior architecture of GPT-4o models. These findings confirm the robustness of our stance detection approach while also emphasizing the influence

of model quality on performance metrics.

2 Validation of topic detection

To validate our topic detection methodology, we utilized the SemEval-2016 Task 6 dataset,[1] which contains tweets labeled for specific topics: Feminist Movement, Hillary Clinton, Legalization of Abortion, and Donald Trump. This dataset provides a ground truth for our validation.

We generated topic dictionaries for the four topics using GPT-4, following the methodology outlined in the Methods. The dictionaries incorporated terms from multiple years, covering various ngrams and both general and Twitter-specific vernaculars, ensuring high recall by capturing the evolving lexicon over the years.

We applied the topic dictionaries to all unique tweets in the SemEval dataset. Each tweet was checked for matches against the terms in the dictionaries. Tweets that matched terms from any dictionary were retained for further processing.

For each topic, we calculated true positives (TP), true negatives (TN), false positives (FP), and false negatives (FN). A true positive occurred when a term from a given topic dictionary matched a tweet labeled for the same topic. For example, if the term "pro-choice" from the "Legalization of Abortion" dictionary matched a tweet labeled as "Legalization of Abortion," it was counted as a TP. A true negative occurred when no term from a given topic dictionary matched tweets labeled for other topics. False positives and false negatives were calculated similarly.

The results in Table 13 show a high recall across topics, demonstrating the effectiveness of our prompt engineering and dictionary generation process. The union of terms across years, ngrams, and vernaculars ensured high recall by capturing a broad range of context-aware expressions

for each topic. This balance between precision and recall demonstrates the robustness of our approach, which captures a wide range of relevant tweets and enhances accuracy.

While these topics are not the same as those used in the main study (except for abortion), the consistent performance across metrics improves our confidence in the external validity of our methodology to other topics. This validation underscores the effectiveness of our method in accurately detecting topics in large tweet datasets, providing a reliable foundation for subsequent stance detection and analysis.

The method is also scalable and cost-efficient, as it requires researchers to create context-aware topic dictionaries and apply these dictionaries to the dataset, rather than parsing each individual document through the LLM.

3 Validation of gender classification

To validate our gender classification method, we used a dataset that combines raw counts for first/given names of male and female babies over various time periods and calculates a probability for each name based on the aggregate count. The source datasets are from reliable government authorities: the US Social Security Card Applications (1880 to 2019), the UK Baby Names in England and Wales Statistical Bulletins (2011 to 2018), the British Columbia 100 Years of Popular Baby Names (1918 to 2018), and the Australian Popular Baby Names from the Attorney-General's Department (1944 to 2019). This extensive dataset with 147,269 unique names will ensure a robust validation process. These datasets were accessed through the UC Irvine Machine Learning Repository `https://archive.ics.uci.edu/dataset/591/gender+by+name`.

We used the OpenAI GPT-3.5-turbo model to predict the gender associated with each name in the validation dataset. Each name was submitted as a prompt to the model, and the responses

were categorized as 'Male', 'Female', or 'Unclear'. The model's predictions were then evaluated against the known genders in the dataset.

The overall unweighted evaluation results are as follows: a precision of 0.8097, a recall of 0.8202, an F1 score of 0.8149, and an accuracy of 0.8610. For the weighted evaluation by count, which gives more importance to names with higher counts, the results are: a precision of 0.9866, a recall of 0.9871, an F1 score of 0.9868, and an accuracy of 0.9863. These metrics, rounded to four decimal places, are summarized in Supplementary Table 14.

These results demonstrate that the gender classification method performs well on a diverse and reliable validation dataset. The high precision, recall, F1 score, and accuracy, particularly in the count-weighted evaluation, instill confidence in the robustness of our classification method.

4 Comparison with opinion poll

To validate the accuracy of the stance metrics derived from Twitter, we compare them with data from the General Social Survey (GSS) for the years 2012, 2014, 2016, 2018, 2021, and 2022. The GSS collects aggregate data across nine regions in the US, with an average sample size of approximately 2,800 respondents per region. For validation, we aggregate the Twitter topic-wise stance metrics at the regional level for each year and compare them with equivalent topic stances from the GSS. In this comparison, the Twitter dataset used is a random sample of approximately 300,000 US users, collected at the same period as the data for academics used in this paper and processed in exactly the same way. The random sample of US users would be representative of the US Twitter population, making the comparison with GSS more meaningful. The aim would be to see whether our stance measures for a Twitter sample of a country have any correlation with the stance measures for a general sample of that country.

While the GSS offers a wealth of respondent data, directly correlating its questions with Twitter metrics can be challenging. This is primarily because GSS questions often encompass multiple dimensions. For example, consider the GSS question on environmental spending: *Are we spending Too Much, Too Little, or About Right on improving and protecting the environment?*. While we can approximate the GSS stance on environmental issues as $GSS_u^{(environment)} = \frac{TooLittle_u - TooMuch_u}{TooLittle_u + TooMuch_u + AboutRight_u}$, this formulation is only a rough equivalent of our net stance metric on Climate Action from Twitter, $S_u^{(t)} = \frac{pro_u^{(t)} - anti_u^{(t)}}{pro_u^{(t)} + anti_u^{(t)} + neutral_u^{(t)}}$, where $t = ClimateAction$. The GSS question not only captures attitudes towards environmental action but also perceptions of current spending levels, introducing a potential bias in the comparison.

To illustrate the relationship between the Twitter and GSS stances, we present scatter plots with lines of best fit for each topic, accompanied by correlation coefficients. Each data point is colour-coded to represent different years. For example, Supplementary Figure 22 displays the relationship for the Climate Action stance. A similar approach is used in Supplementary Figure 23 for the topic of Abortion Rights (this being a common policy issue in US). Interestingly, the Twitter and GSS stance metrics for Abortion Rights exhibit a stronger positive correlation compared to Climate Action. This stronger correlation may stem from the more straightforward nature of the GSS question on abortion rights, which focuses solely on the respondent's social values: *Please tell me whether or not you think it should be possible for a pregnant woman to obtain a legal abortion if the woman wants it for any reason*. The GSS stance on abortion rights is calculated as $GSS_u^{AbortionRights} = \frac{Yes_u - No_u}{Yes_u + No_u}$.

5 Additional Results

5.1 Granular Field Level Distribution

The match of Twitter data with OpenAlex allows us to go very granular in differences across fields. In this paper we show the distribution across 19 root level concepts like Physics, Economics and Geography. We rank academic disciplines by the median level of egocentric expression and also provide the 25th and 75th percentile to hint at distribution.

Supplementary Figure 1 shows the field level distributions for **Egocentrism**. The disciplines of Physics, Philosophy, and Mathematics exhibit the highest levels of egocentrism, while fields like Business, Engineering and Medicine are at the lower end.

Supplementary Figure 2 shows that support for **Climate Action** is strongest within the sciences, specifically Mathematics, Physics, Material Sciences, and weakest in Economics, Sociology and Political Science. This is alarming given that policy relevant research typically produced in these social sciences.

Supplementary figures 3 and 4 provide distributions for two measures: **Behavioral Adjustment** and **Techno Optimism**. The distributions make intuitive sense: the highest support for techno-optimistic solutions in fields like Materials Science, Chemistry, Geology, Environmental Science, and Engineering, whereas Mathematics and Psychology lean more towards behavioral adjustments.

Supplementary Figures 5 and 6 show how expressed stances on socio-economic issues vary by fields. Cultural Liberalism is always positive on average, with variation in intensity. Chemistry scholars most culturally liberal, while Economics and Business are on the bottom. Economic Collectivism always has a positive median, but 25th percentiles can often be net negative. Lowest levels of support for economic collectivism is within Engineering and Economics, while highest

are in Psychology and Physics.

5.2 Correlates of Egocentric Expression

There would be some natural relationships between these expressions. We saw certain subgroups score similarly on certain metrics, yet there were differences on others. Supplementary Figures 7 and 8 depict correlates of Egocentric and Political Expression (respectively), relative to other expressions. These are coefficients of a fixed effects model that control for any unobserved characteristics that are constant over time for time-period (year by month). In panel (a), we see Toxicity, Emotionality, Cultural Liberalism and Economic Collectivism to be positive predictors of egocentric expression. Egocentric expression is negatively correlated with Climate Action, Techno-Optimism and with University Rankings. There is a statistically insignificant relationship with Behavioural Adjustment and Impact Factor.

Panel (b) shows correlates of political expression. Pro-climate action expressions are associated with less toxicity, higher impact factor, higher university rankings and more lower emotionality of expression. Cultural liberalism, but not economic collectivism, is associated with more toxic and emotional speech.

We emphasise the limitation of this exercise: both dependent and independent variables are text-based measures from social media, hence there is risk of classical measurement error. This is less of an issue in other sections, where the text-based measure is generally only the dependent variable.

5.3 Robustness of Subgroup Differences in Political Stances After Controlling for Tone and Style Variables

In this section, we examine whether subgroup differences in political stances are confounded by variations in tone and style of expression. Specifically, we investigate three expression styles—emotionality, egocentrism, and toxicity—by first examining their raw correlations with political stances and then running regressions controlling for each of these expression styles. This helps us verify whether the "how" of expression impacts the subgroup differences in the "what" of academic expression.

Raw Correlations Between Stances and Expression Styles We begin by examining the raw correlations between the five stances—*Climate Action*, *Techno-Optimism*, *Behavioural Adjustment*, *Cultural Liberalism*, and *Economic Collectivism*—and three expression styles—*Emotionality*, *Egocentrism*, and *Toxicity*.

The results in Supplementary Table 7 show that the correlation between stances and expression styles is very close to zero in most cases, suggesting minimal direct relationships between how academics express themselves and the stances they express. The highest correlation observed is 0.085 ($r = 0.085$, $p < 0.0001$, 95% CI [0.0804, 0.0904]) for *Cultural Liberalism* and *Toxicity*, still relatively low. This suggests that tone and style of expression are unlikely to confound the subgroup differences we observe in stance.

Regression Analysis Controlling for Expression Styles Next, we investigate whether expression styles confound subgroup differences in political stances. To do this, we run regressions where we compare subgroup differences while controlling for one of the expression styles (emotionality, egocentrism, or toxicity) at a time. Specifically, we estimate the following model:

$$\text{StanceExpression}_{it} = \alpha + \beta_1 \text{GroupVar}_{it} + \beta_2 \text{HowExpression}_{it} + \epsilon_{it}$$

Where GroupVar_{it} represents a subgroup indicator (e.g., gender, field, rankings, or country), and $\text{HowExpression}_{it}$ represents one of the three expression styles (emotionality, egocentrism, or toxicity). This allows us to assess whether controlling for the "how" variables alters the subgroup coefficient β_1. If β_1 remains stable across the three models, it indicates that tone and style of expression do not confound subgroup differences.

Climate-Related Stances The results in Supplementary Figure 9 show that the subgroup differences in climate-related stances are robust across all three regressions. Whether we control for emotionality, egocentrism, or toxicity, the subgroup coefficients remain qualitatively unchanged, suggesting that expression styles do not confound these differences.

Socio-Economic Stances As in the case of climate-related stances, Supplementary Figure 10 demonstrates that the subgroup differences in cultural and economic stances are robust to controls for emotionality, egocentrism, and toxicity. The coefficients for subgroup indicators remain stable across all regressions.

This analysis shows that tone and style of expression—whether emotionality, egocentrism, or toxicity—do not confound the observed subgroup differences in stances. The qualitative patterns of subgroup differences hold across all three models, suggesting that these differences are driven by the "what" of academic expression, not the "how." This finding supports the robustness of our main results and confirms that the observed subgroup differences in stances reflect genuine variation in academic expression across demographic and professional groups.

5.4 Robust Trends - Partialling Out Individual Fixed Effects

To uncover the overarching trends in a behavioural or political expressions, we isolate individual author level fixed effects. This methodological choice allows us to isolate and examine the temporal dynamics of an expression, controlling for the inherent heterogeneity across authors. The model is mathematically represented as follows:

$$\text{Expression}_{it} = \lambda_t \times \text{Time}_t + \mu_i + \epsilon_{it} \quad (2)$$

where Expression_{it} measures the expression of a political or behavioural identity by author i at time t. This includes an expression of net stance or narrative. α represents the intercept, λ_t captures the effect of time, categorized by year and month, on Expression, μ_i is the author-specific fixed effects, and ϵ_{it} is the error term. The inclusion of author-specific fixed effects enables us to partial out the influence of individual characteristics that are consistent over time, thus ensuring that the identified trends in Y are not confounded by these idiosyncratic factors.

This approach is particularly advantageous for several reasons. First, it acknowledges the heterogeneity inherent in academic behaviors, recognizing that individual authors may exhibit varying levels of expression based on personal, disciplinary, or topical engagement factors. By controlling for individual author-level fixed effects, we isolate the temporal trends in political expression while accounting for these variations. Second, by controlling for these author-specific effects, we can more accurately isolate the influence of time on Expression, yielding insights that are more reflective of true temporal trends rather than artifacts of the composition of the author cohort. Lastly, this method lays the groundwork for future research to explore the underlying causes of these trends, offering a clearer path for investigating the factors that drive variations in academic expression.

Supplementary Figures 18–21 present the within-individual trends for the overall sample and various subgroups, including gender (Supplementary Figure 19), field of study (Supplementary Figure 20), and country of institution (Supplementary Figure 21). These figures allow for a comparison of temporal patterns within subgroups while controlling for individual fixed effects. When comparing the within-user trends in these Supplementary figures to the raw trends presented earlier (e.g., Figure 7 and Supplementary Figures 11–13), we observe that the overall patterns appear similar. For instance, the trends in egocentrism, toxicity, and emotionality over time in the within-user analyses resemble those in the raw data for different subgroups.

In Supplementary Figure 19, the within-user trends by gender illustrate the temporal dynamics for male and female academics. Similarly, Supplementary Figure 20 displays the within-user trends by field, showing patterns for scholars in humanities, social sciences, and STEM fields. Supplementary Figure 21 presents the within-user trends for academics based in the U.S. versus non-U.S. institutions. By comparing these within-user trends with their corresponding raw trends, we can assess the consistency of the temporal patterns across different analytical approaches. The similarities between the two sets of figures suggest that the observed trends are not solely due to changes in the composition of individuals active on social media over time but also reflect within-individual changes. Overall, these within-user trends support the robustness of our findings and provide additional insights into the temporal dynamics of academic expression across various subgroups.

6 Tables

Table 1: Power-Law Fit Parameters for Academic Influence Metrics

Metric	Power-Law Exponent (α)	Minimum Value (x_{min})	KS Statistic	Log-Likelihood Ratio
Content Creation (Posts)	3.337	2650	0.007	-1.112 (p = 0.399)
Engagement (Likes Received)	3.146	15491.5	0.015	-3.660 (p = 0.112)
Public Reach (Followers)	2.636	1332	0.011	0.403 (p = 0.001)
Citations Count	2.929	15808	0.033	-12.281 (p = 0.003)

This table summarizes the results of fitting a power-law distribution to various metrics of academic influence on Twitter: content creation (posts), engagement (likes received), public reach (followers), and citations count. The analysis follows the method by Claueset et al (2009),[3] estimating the power-law exponent (α) and the minimum value (x_{min}) above which the data follows a power-law distribution. The 95% confidence intervals (CI) for α are provided. The Kolmogorov-Smirnov (KS) statistic measures the goodness of fit, with lower values indicating a better fit to the power-law model. The p-value corresponds to the goodness-of-fit test, where higher values suggest the power-law is a plausible fit. The results confirm that these metrics exhibit power-law behavior, indicating significant inequality across these academic influence measures.

Table 2: Normality and Variance Homogeneity Checks for Climate Action and Climate Narrative Comparisons

**Comparison**	**Sample Sizes** (N_1, N_2)	**Skewness** (G1, G2)	**Kurtosis** (G1, G2)	**Levene's F** (df_1, df_2)	**Levene's *p***
Behavioral Adjustment vs. Techno Optimism	151,791, 151,791	3.067, 4.137	10.409, 18.116	1221.43 (1, 303,582)	< 0.001
Academics vs. General Users (Climate Action)	51,633, 3,488,181	2.507, 7.226	10.609, 97.95	26,390.01 (1, 3,539,812)	< 0.001
Academics vs. General Users (Techno-Optimism)	51,633, 3,488,181	4.43, 2.677	20.623, 8.57	1,544.11 (1, 127,626)	< 0.001
Male vs. Female Academics (Climate Action)	85,117, 66,675	2.17, 2.42	8.79, 9.18	12.13 (1, 151,790)	< 0.001
Male vs. Female Academics (Techno-Optimism)	85,117, 66,675	3.65, 5.07	14.32, 26.67	560.60 (1, 151,790)	< 0.001
STEM vs. Social Sciences (Climate Action)	84,594, 50,274	2.16, 2.52	7.98, 11.22	260.84 (1, 134,866)	< 0.001
STEM vs. Humanities (Techno Optimism)	84,594, 2,355	3.71, 5.19	14.78, 27.98	13.67 (1, 85,700)	< 0.001
Academics (High Reach, Low Credibility) vs. Experts (Climate Action)	8,964, 7,465	2.971, 2.944	12.194, 9.666	3,640.93 (1, 3,497,143)	< 0.001
U.S. Based vs. Non U.S. Academics (Behavioral Adjustment)	38,666, 113,126	3.503, 2.944	13.269, 9.666	164.59 (1, 151,790)	< 0.001
Top 100 vs. 101–500 Universities (Climate Action)	30,134, 93,616	2.558, 2.185	20.185, 23.998	3.06 (1, 123,748)	0.081

This table reports the results of normality and variance homogeneity checks for group comparisons related to Climate Action and Climate Narratives (Behavioral Adjustment and Techno-Optimism). Skewness and kurtosis values (G_1, G_2) assess normality for each group, while Levene's test evaluates the homogeneity of variances. Significant Levene's test results ($p < 0.05$) indicate heterogeneity of variances, for which Welch's t-test is applied in subsequent analyses. Sample sizes, degrees of freedom, and Levene's F-statistic with exact p-values are reported for each comparison. See Methods for detailed statistical procedures.

Table 3: Normality and Variance Homogeneity Checks for Cultural Liberalism and Economic Collectivism Comparisons

**Comparison**	**Sample Sizes** (N_1, N_2)	**Skewness** (G1, G2)	**Kurtosis** (G1, G2)	**Levene's F** (df_1, df_2)	**Levene's *p***
Academics vs. U.S. Twitter Users (Cultural Liberalism)	51,633, 3,488,181	0.59, 3.801	4.306, 40.675	76,889.05 (1, 3,539,812)	< 0.001
Academics vs. U.S. Twitter Users (Economic Collectivism)	51,633, 3,488,181	2.549, 2.536	23.532, 191.105	22,479.83 (1, 3,539,812)	< 0.001
Non-Experts vs. Experts (Cultural Liberalism)	137,752, 14,040	0.873, 0.711	5.453, 4.705	93.20 (1, 151,790)	< 0.001
Non-Experts vs. Experts (Economic Collectivism)	139,740, 12,052	2.56, 2.10	24.60, 17.83	77.47 (1, 151,790)	< 0.001
Female vs. Male Academics (Cultural Liberalism)	66,675, 85,117	0.738, 0.957	4.759, 5.985	514.52 (1, 151,790)	< 0.001
Female vs. Male Academics (Economic Collectivism)	66,675, 85,117	2.577, 2.461	22.912, 24.712	14.63 (1, 151,790)	< 0.001
Social Scientists vs. Non-Social Scientists (Economic Collectivism)	50,274, 101,518	2.444, 2.549	21.011, 25.615	79.81 (1, 151,790)	< 0.001
Social Scientists vs. Non-Social Scientists (Cultural Liberalism)	50,274, 101,518	0.817, 0.879	5.373, 5.372	5.30 (1, 151,790)	0.021
Humanities vs. Non-Humanities (Cultural Liberalism)	2,355, 149,437	0.608, 0.862	4.452, 5.392	17.60 (1, 151,790)	< 0.001
Humanities vs. Non-Humanities (Economic Collectivism)	2,355, 149,437	1.997, 2.527	20.364, 23.951	0.79 (1, 151,790)	0.375
Top 100 vs. 101–500 Universities (Cultural Liberalism)	30,134, 32,661	0.728, 0.900	4.775, 5.658	109.06 (1, 62,793)	< 0.001
Top 100 vs. 101–500 Universities (Economic Collectivism)	30,134, 32,661	2.558, 2.185	23.689, 23.998	0.16 (1, 62,793)	0.688
U.S.-Based vs. Non-U.S. Academics (Cultural Liberalism)	38,666, 113,126	0.588, 0.968	4.286, 5.898	982.18 (1, 151,790)	< 0.001
U.S.-Based vs. Non-U.S. Academics (Economic Collectivism)	38,666, 113,126	2.690, 2.465	24.586, 23.646	6.97 (1, 151,790)	0.008

This table summarizes normality and variance homogeneity checks for group comparisons related to Cultural Liberalism and Economic Collectivism. Normality is assessed using skewness and kurtosis values (G_1, G_2), while Levene's test evaluates homogeneity of variances. Significant results ($p < 0.05$) suggest unequal variances, leading to the application of Welch's t-test for mean comparisons. Sample sizes, degrees of freedom, and Levene's F-statistic with exact p-values are provided. Refer to Methods for an explanation of the statistical framework.

Table 4: Normality and Variance Homogeneity Checks for Behavioral Feature Comparisons

**Comparison**	**Sample Sizes** (N_1, N_2)	**Skewness** (G1, G2)	**Kurtosis** (G1, G2)	**Levene's F** (df_1, df_2)	**Levene's *p***
Humanities vs. Others (Egocentrism)	2,355, 149,437	2.31, 4.67	14.68, 111.81	0.25 (1, 151,790)	0.618
Social Sciences vs. Others (Egocentrism)	50,274, 101,518	2.58, 5.36	18.86, 139.15	77.15 (1, 151,790)	< 0.001
High Reach, Low Credibility vs. Others (Egocentrism)	44,735, 107,057	3.01, 5.09	46.67, 125.56	77.15 (1, 151,790)	< 0.001
Low Reach, High Credibility vs. Others (Egocentrism)	24,463, 127,329	6.05, 4.20	162.02, 91.09	103.64 (1, 151,790)	< 0.001
Top 100 vs. 101–500 Universities (Egocentrism)	30,134, 32,661	2.56, 2.34	20.19, 17.06	101.73 (1, 62,793)	< 0.001
Top 100 vs. 501–1500 Universities (Egocentrism)	30,134, 10,704	2.56, 2.03	20.19, 11.18	30.92 (1, 40,836)	< 0.001
U.S.-Based vs. Non-U.S. (Egocentrism)	38,666, 113,126	2.30, 5.60	16.84, 150.84	363.61 (1, 151,790)	< 0.001
Humanities vs. Others (Toxicity)	2,345, 148,804	3.50, 4.20	19.77, 27.65	7.51 (1, 151,147)	0.006
Social Sciences vs. Others (Toxicity)	50,050, 101,099	3.93, 4.32	24.32, 29.21	31.84 (1, 151,147)	< 0.001
STEM vs. Others (Toxicity)	84,243, 66,906	4.30, 4.04	29.31, 25.37	12.75 (1, 151,147)	< 0.001
Low Reach, High Credibility vs. High Reach, Low Credibility (Toxicity)	24,385, 44,521	3.99, 4.24	24.92, 28.03	50.92 (1, 151,147)	< 0.001
Top 100 vs. 101–500 Universities (Toxicity)	30,013, 32,512	4.09, 4.29	26.51, 28.22	18.16 (1, 62,523)	< 0.001
Top 100 vs. 501–1500 Universities (Toxicity)	30,013, 10,660	4.09, 4.25	26.51, 28.13	9.37 (1, 40,671)	0.002
U.S.-Based vs. Non-U.S. (Toxicity)	38,506, 112,643	3.86, 4.31	23.75, 29.08	111.43 (1, 151,147)	< 0.001
Academics vs. U.S. Twitter Users (Emotionality)	51,633, 2,797,593	8.29, 4.04	426.26, 200.15	2,838.04 (1, 2,849,224)	< 0.001
U.S.-Based vs. Non-U.S. (Emotionality)	38,666, 113,126	10.20, 2.65	553.00, 16.17	6.80 (1, 151,790)	0.009
Female vs. Male (Emotionality)	66,675, 85,117	7.02, 2.73	349.83, 18.07	89.92 (1, 151,790)	< 0.001
STEM vs. Non-STEM (Emotionality)	84,594, 67,198	2.57, 7.57	16.09, 390.20	6.53 (1, 151,790)	0.011

This table provides results of normality and variance homogeneity checks for comparisons of behavioral features (**Egocentrism**, **Toxicity**, and **Emotionality**). Skewness and kurtosis values (G_1, G_2) test for normality, while Levene's test evaluates variance homogeneity. Significant Levene's test results ($p < 0.05$) indicate variance heterogeneity, for which Welch's t-test is used in subsequent analyses. Sample sizes, degrees of freedom, Levene's F-statistic, and exact p-values are reported. See Methods for details on the statistical approach.

Table 5: Normality and Variance Homogeneity Checks for Temporal Dynamics in Academic Expression

**Comparison**	**Sample Sizes** (N_1, N_2)	**Skewness** (G1, G2)	**Kurtosis** (G1, G2)	**Levene's F** (df_1, df_2)	**Levene's *p***
Climate Action: Pre- vs. Post-Pandemic (Academics)	28,076, 23,557	2.27, 2.86	9.70, 11.94	48.23 (1, 51,631)	< 0.001
Economic Collectivism: Pre- vs. Post-Pandemic (Academics)	28,076, 23,557	1.99, 3.32	21.92, 25.69	14.67 (1, 51,631)	< 0.001
Climate Action: Pre- vs. Post-Pandemic (U.S. Users)	2,063,027, 1,425,154	7.13, 7.37	95.38, 101.92	38.61 (1, 3,488,179)	< 0.001
Economic Collectivism: Pre- vs. Post-Pandemic (U.S. Users)	2,063,027, 1,425,154	1.79, 2.97	229.61, 153.39	1,395.64 (1, 3,488,179)	< 0.001
Cultural Liberalism: 2020–2022 vs. Other (U.S. vs. Non-U.S.)	18,470, 49,023	0.43, 0.82	3.75, 5.29	757.37 (1, 67,491)	< 0.001

This table details normality and variance homogeneity checks for pre- and post-pandemic comparisons of Climate Action and Economic Collectivism in both academics and general U.S. Twitter users. Skewness and kurtosis values (G_1, G_2) assess normality, while Levene's test examines variance homogeneity. Significant results ($p < 0.05$) indicate heterogeneity of variances, for which Welch's t-test is applied in subsequent analyses. Sample sizes, degrees of freedom, Levene's F-statistic, and exact p-values are included. See Methods for an overview of the statistical framework.

Table 6: Normality and Variance Homogeneity Checks for Comparisons of Academic vs. Non-Academic Users

**Comparison**	**Sample Sizes** (N_1, N_2)	**Skewness** (G1, G2)	**Kurtosis** (G1, G2)	**Levene's F** (df_1, df_2)	**Levene's *p***
Egocentrism: Academics vs. General U.S. Twitter (2022)	6,853, 362,033	2.55, 3.00	22.03, 20.57	476.61 (1, 368,884)	< 0.001
Toxicity: Academics vs. General U.S. Twitter	51,432, 1,644,511	3.84, 3.05	23.57, 13.85	3,241.56 (1, 1,695,941)	< 0.001
Emotionality: Academics vs. General U.S. Twitter	51,633, 2,797,593	8.29, 4.04	426.26, 200.15	2,838.04 (1, 2,849,224)	< 0.001
Climate Action: Academics (Pre- vs. Post-Pandemic)	28,076, 23,557	2.27, 2.86	9.70, 11.94	48.23 (1, 51,631)	< 0.001
Economic Collectivism: Academics (Pre- vs. Post-Pandemic)	28,076, 23,557	1.99, 3.32	21.92, 25.69	14.67 (1, 51,631)	< 0.001
Climate Action: General U.S. Twitter (Pre- vs. Post-Pandemic)	2,063,027, 1,425,154	7.13, 7.37	95.38, 101.92	38.61 (1, 3,488,179)	< 0.001
Economic Collectivism: General U.S. Twitter (Pre- vs. Post-Pandemic)	2,063,027, 1,425,154	1.79, 2.97	229.61, 153.39	1,395.64 (1, 3,488,179)	< 0.001
Climate Action: U.S. vs. Non-U.S. Academics (2020–2022)	18,470, 49,023	0.43, 0.82	3.75, 5.29	757.37 (1, 67,491)	< 0.001
Cultural Liberalism: U.S. vs. Non-U.S. Academics (2020–2022)	18,470, 49,023	0.43, 0.82	3.75, 5.29	757.37 (1, 67,491)	< 0.001

This table reports normality and variance homogeneity checks for comparisons of academic and non-academic users across behavioral features (**Egocentrism**, **Toxicity**, **Emotionality**), Climate Action, and Economic Collectivism. Skewness and kurtosis values (G_1, G_2) assess normality for each group, while Levene's test evaluates variance homogeneity. Significant Levene's test results ($p < 0.05$) indicate heterogeneity of variances, warranting the use of Welch's t-test for subsequent analyses. Sample sizes, degrees of freedom, Levene's F-statistic, and exact p-values are provided. Refer to Methods for statistical details.

Table 7: Raw Correlations Between Stances and Emotionality, Egocentrism, and Toxicity

**Stance**	**Emotionality**	**Egocentrism**	**Toxicity**
Climate Action	-0.0081	-0.0272	-0.0326
	p = 0.0017	*p = 2.5750e-26*	*p = 6.6847e-37*
	[-0.0131, -0.0030]	[-0.0323, -0.0222]	[-0.0377, -0.0276]
Techno-Optimism	-0.0166	-0.0407	-0.0553
	p = 9.3587e-11	*p = 9.2021e-57*	*p = 8.7137e-103*
	[-0.0217, -0.0116]	[-0.0458, -0.0357]	[-0.0604, -0.0503]
Behavioural Adjustment	-0.0075	0.0003	-0.0135
	p = 0.0035	*p = 9.1285e-01*	*p = 1.4721e-07*
	[-0.0125, -0.0025]	[-0.0047, 0.0053]	[-0.0186, -0.0085]
Cultural Liberalism	0.0209	0.0568	0.0854
	p = 4.0620e-16	*p = 9.6633e-109*	*p = 1.9624e-242*
	[0.0159, 0.0259]	[0.0518, 0.0618]	[0.0804, 0.0904]
Economic Collectivism	0.0035	0.0149	0.0002
	p = 1.7271e-01	*p = 5.9204e-09*	*p = 9.2672e-01*
	[-0.0015, 0.0085]	[0.0099, 0.0200]	[-0.0048, 0.0053]

Each cell shows the Pearson correlation coefficient (r) between a given stance (rows) and one of three metrics of expression style (columns): **Emotionality** (ratio of affective to cognitive words), **Egocentrism** (prevalence of first-person singular pronouns), and **Toxicity** (Google's Perspective API). Parenthetical 95% confidence intervals were computed via Fisher's z-transformation. All tests are two-sided, and exact p-values are reported without any multiple-comparison adjustments. The sample is a panel dataset comprising $n = 151{,}792$ academic-month observations, and degrees of freedom are $df = n - 2$. Correlations are small in magnitude (largest $|r| \approx 0.085$), indicating minimal direct association between expression style (*how* academics communicate) and stance (*what* academics express).

Table 8: Tweet level Summary Statistics

	Mean	Median	SD	Min	P25	P75	Max	N
**Full Sample**								
N. Words	18.14	16	12.00	0	9	24	141	138,372,165
N. Characters	140.11	127	79.18	0	79	179	1,141	138,372,165
N. Likes	4,525	2,701	6,956	0	1,181	5,290	293,797	138,372,165
N. Retweets	614.13	1	14,889	0	0	7	3,712,839	138,372,165
==1 if post is a Retweet	0.42	0	0.49	0	0	1	1	138,372,165
==1 if post is in English	0.77	1	0.42	0	1	1	1	138,372,165
**English Tweets Sample**								
N. Words	19.49	17	11.68	0	11	26	122	94,064,897
N. Egocentric Words	0.33	0	0.77	0	0	0	36	94,064,897
N. Affective Words	1.22	1	1.36	0	0	2	56	94,064,897
N. Cognition Words	2.58	2	2.09	0	1	3	71	94,064,897
Share Egocentric Words	0.02	0	0.04	0	0	0	1	94,064,897
Share Affective Words	0.06	0.05	0.08	0	0	0.10	1	94,064,897
Share Cognition Words	0.14	0.13	0.12	0	0.07	0.19	1	94,064,897
Toxicity probability	0.04	0.02	0.07	0	0.01	0.03	0.98	12,698,961

This table presents tweet-level summary statistics from a dataset of 138,372,165 tweets by academics between January 1, 2016, and December 31, 2022. The table is divided into two parts: the full sample and a subset of 94,064,897 English tweets. Metrics include the number of words, characters, likes, retweets, and indicators for retweets and English language. Affective and cognitive word frequencies, are calculated using the Linguistic Inquiry and Word Count (LIWC) dictionary.[4] Egocentric words include "I," "me," "my," "myself," and "mine." Shares of egocentric, affective, and cognitive words are computed relative to the total number of words per tweet. Toxicity probabilities, derived using Google's Perspective API, measure the likelihood of harmful or aggressive language. For toxicity, due to API rate limits, we randomly sampled up to 10 English tweets per academic per month. Refer to Methods for detailed descriptions of data processing and variable definitions.

Table 9: Balancedness Test: Comparison of Balanced and Unbalanced Populations

Panel (a): Balanced Population						
**Variable**	**Mean**	**Median**	**SD**	**Min**	**Max**	**N**
**Publication Metrics**						
Nbr. Citations	1370.02	213	4523.99	1	238736	99274
Impact Factor (2Y)	16.93	9	38.56	0.02	4205	99274
Nbr. Works	57.58	24	114.38	1	7711	99274
**Demographics**						
Humanities	0.005	0	0.07	0	1	99274
STEM	0.291	0	0.45	0	1	99274
Social Sciences	0.098	0	0.30	0	1	99274
Gender: Male	0.60	1	0.49	0	1	99274
**Twitter Metrics**						
Nbr. Likes	3210.85	1673	5736.33	0	293797	99274
Nbr. Followers	1112.70	533	9145.94	0	1087504	99274
Nbr. Accounts Followed	749	529	1116.55	0	191923	99274
Nbr. Retweets	843.72	276	2117.91	0	181268	99274
Nbr. Posts	1575.24	1032	2052.13	4	117088	99274
**Panel (b): Unbalanced Population**						
**Variable**	**Mean**	**Median**	**SD**	**Min**	**Max**	**N**
**Publication Metrics**						
Nbr. Citations	1083.30	139	3969.42	1	331147	219273
Impact Factor (2Y)	15.49	9	30.96	0.02	2539	219273
Nbr. Works	48.26	18	110.51	1	8465	219273
**Demographics**						
Humanities	0.002	0	0.04	0	1	219273
STEM	0.262	0	0.44	0	1	219273
Social Sciences	0.071	0	0.26	0	1	219273
Gender: Male	0.59	1	0.49	0	1	219273
**Twitter Metrics**						
Nbr. Likes	5909.80	847	18705.81	0	2017914	219150
Nbr. Followers	2464.89	264	272177.95	0	126938095	219150
Nbr. Accounts Followed	713.74	328	2176.71	0	382058.5	219150
Nbr. Retweets	732.73	141	5519.14	0	1513703	219150
Nbr. Posts	3639.87	293	12652.17	1	734460	219150

This table compares author-level summary statistics between two populations of academics on Twitter: the balanced sample (**Panel a**) and the unbalanced sample (**Panel b**). The balanced sample (n = 99,274) includes individuals who tweeted at least once in both the first six months of 2016 and the last six months of 2022, ensuring consistent presence over the entire study period. The unbalanced sample (n = 219,273) consists of individuals who tweeted at least once at any point during 2016–2022, regardless of activity consistency. Metrics are presented across three categories: publication metrics (e.g., citations, works, impact factor), demographics (e.g., gender, field of study), and Twitter metrics (e.g., likes, followers, posts). Summary statistics include the mean, median, standard deviation (SD), minimum, and maximum values for each variable. Refer to Methods for a detailed description of the sampling strategy and variable definitions.

Table 11: Evaluation Metrics for Stance Detection

Task	Target	GPT 3.5 Turbo (F_{avg})	GPT 4 (F_{avg})
A	Feminism	92.44	81.89
A	Hillary Clinton	89.57	87.53
A	Abortion	79.52	84.36
B	Donald Trump	84.18	80.00

This table reports the F_{avg} scores (Supplementary Equation 1) for stance detection tasks using GPT-3.5 Turbo and GPT-4. Validation was conducted using 40,317 hand-labeled tweets from the ACM SemEval-2016 Task 6 dataset.[1] Each task involves predicting stances (pro, anti, neutral) toward targets such as Feminism, Hillary Clinton, and Abortion. Results indicate strong performance, with F_{avg} scores ranging from 79.52 to 92.44 for GPT-3.5 Turbo and 80.00 to 87.53 for GPT-4. Higher F_{avg} scores denote better alignment with human-labeled stances, highlighting the accuracy of the GPT-based stance detection methodology.

Table 12: Comparison of Agreement and F1 Scores Across GPT Models

Comparison	Agreement (Modal)	Agreement (Iterations)	F1 (Modal)	F1 (Iterations)
GPT-3.5-turbo vs GPT-4o	0.781	0.772	0.806	0.795
GPT-3.5-turbo vs GPT-4	0.750	0.738	0.772	0.756
GPT-3.5-turbo vs GPT-4o-mini	0.684	0.681	0.696	0.691

This table compares agreement rates and average F_{avg} scores (Supplementary Equation 1) between different GPT models for stance detection. Agreement metrics include: **Modal Agreement**, the proportion of identical stance predictions when using the modal stance across 10 iterations per tweet, and **Iteration Agreement**, which measures agreement across individual iterations. F_{avg} scores assess precision and recall consistency, with higher values indicating better model alignment. Results highlight strong consistency across models, particularly between GPT-3.5 Turbo and GPT-4o ($F_{avg} = 0.806$ for Modal Agreement). These findings demonstrate the robustness of GPT-based stance detection, even across variations in model architecture.

7 Figures

Figure 1: Distribution of **Egocentric** Expressions by field

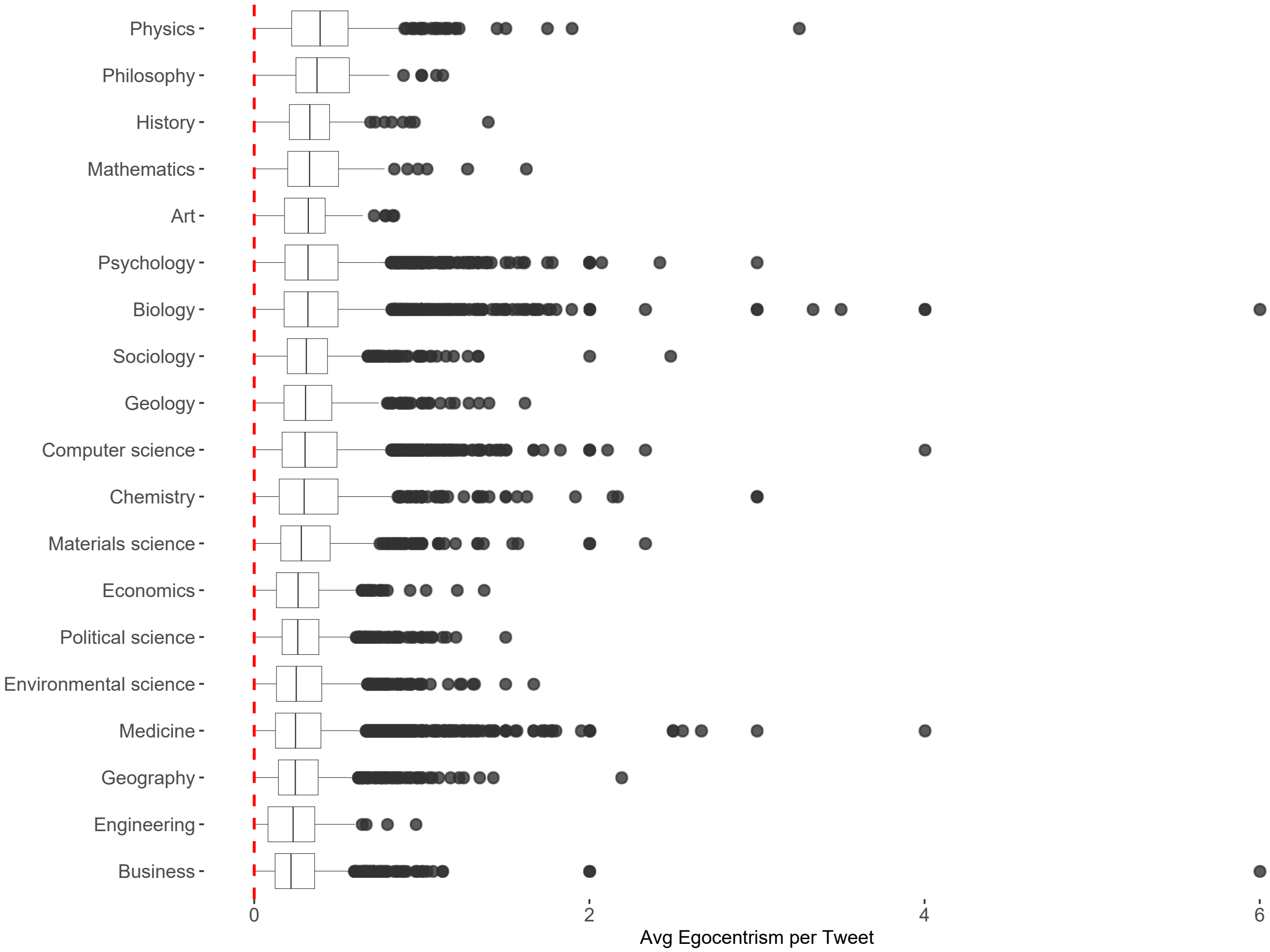

Data come from **n** = **99,274** academics in a balanced sample (2016–2022). For each academic, *egocentrism* is computed as the mean number of first-person singular terms ("I," "me," "my," "myself," "mine") per English tweet. These per-academic means are shown in a box plot by *field*, ordered in descending median. Center lines indicate the median value; boxes span the 25th–75th percentiles (IQR). Whiskers extend to 1.5 times the IQR from each hinge. Hence, the box depicts the middle 50% of authors in each field.

Figure 2: Distribution of **Climate Action** Expression by field

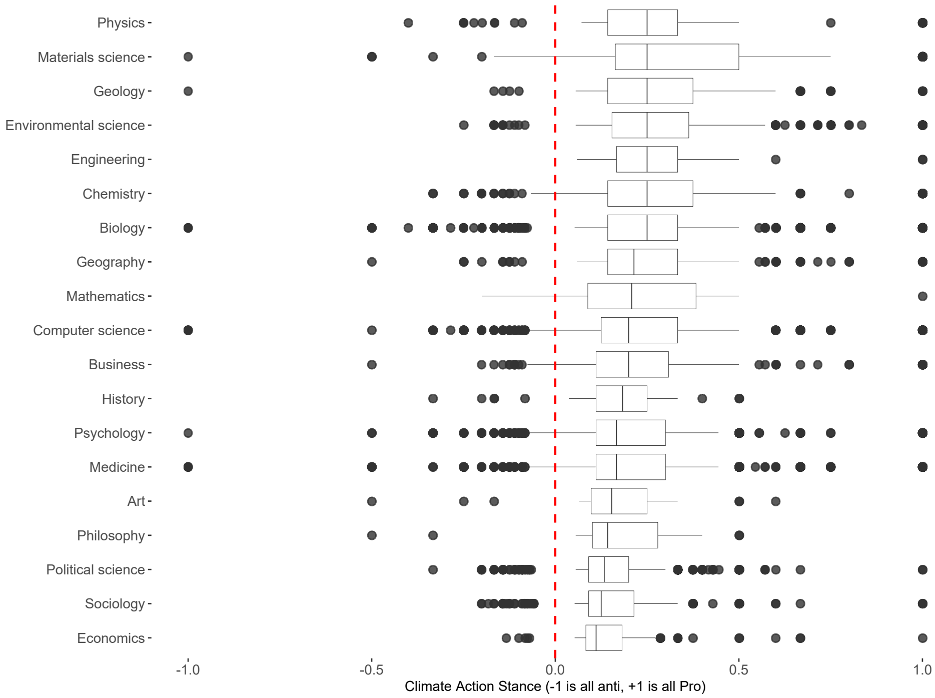

Data come from $\mathbf{n} = \mathbf{99{,}274}$ academics in a balanced sample (2016–2022), but here we focus on those with a *non-zero* ($\neq 0$) **Climate Action** stance (i.e. not neutral), with resulting sample size of $\mathbf{n} = \mathbf{21{,}747}$ academics. Zero-stances are excluded to emphasize distributions among authors who expressed at least some positive or negative position. Each box plot shows the distribution of per-academic mean stance (ranging from -1 = fully anti to +1 = fully pro), grouped by *field* and ordered in descending median stance. Center lines mark the median; boxes represent the IQR; whiskers extend 1.5 IQR beyond the hinges. A vertical red dashed line at $x = 0$ denotes neutral stance for reference.

Figure 3: Distribution of **Behavioural Adjustment** Narrative in Climate Action by field

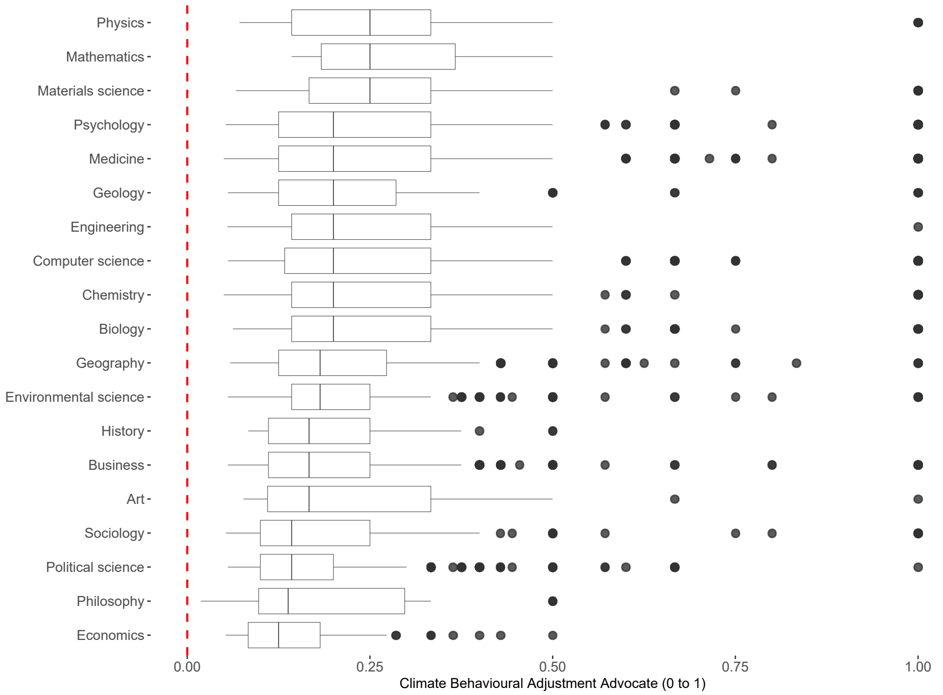

Data come from **n** = **99,274** academics in a balanced sample (2016–2022), but here we focus on those with a *non-zero* ($\neq 0$) **Behavioural Adjustment** stance (i.e. not neutral), with resulting sample size of **n** = **4,612** academics. Zero-stances are excluded to emphasize distributions among authors who expressed at least some positive or negative position. Each box plot shows the distribution of per-academic mean stance (ranging from -1 = fully anti to +1 = fully pro), grouped by *field* and ordered in descending median stance. Center lines mark the median; boxes represent the IQR; whiskers extend 1.5 IQR beyond the hinges. A vertical red dashed line at $x = 0$ denotes neutral stance for reference.

Figure 4: Distribution of **Techno-Optimism** Narrative in Climate Action by field

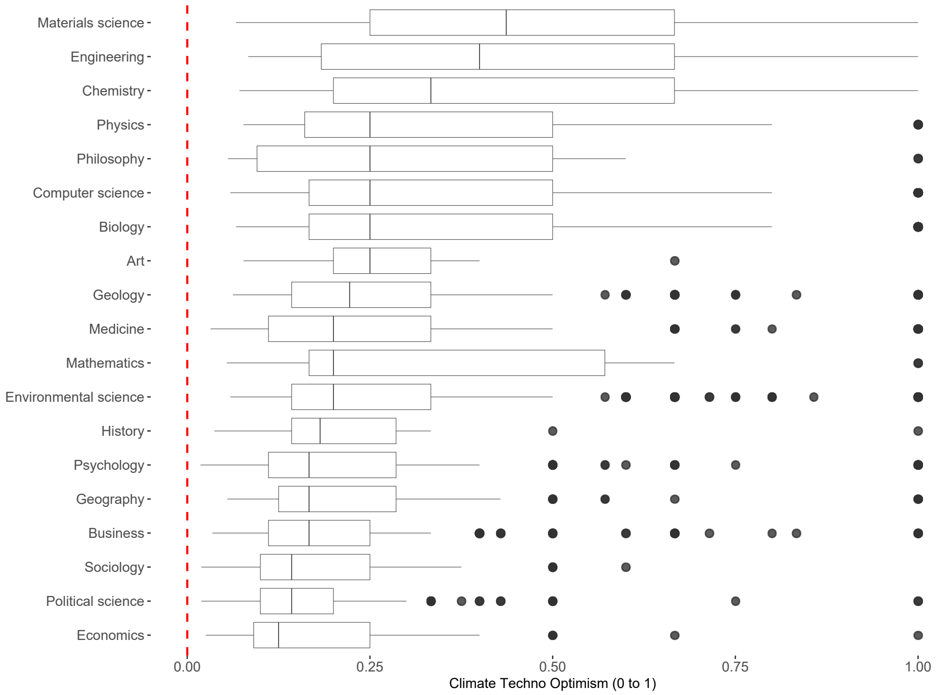

Data come from $\mathbf{n} = \mathbf{99{,}274}$ academics in a balanced sample (2016–2022), but here we focus on those with a *non-zero* ($\neq 0$) **Techno-Optimism** stance (i.e. not neutral), with resulting sample size of $\mathbf{n} = \mathbf{11{,}780}$ academics. Zero-stances are excluded to emphasize distributions among authors who expressed at least some positive or negative position. Each box plot shows the distribution of per-academic mean stance (ranging from -1 = fully anti to +1 = fully pro), grouped by *field* and ordered in descending median stance. Center lines mark the median; boxes represent the IQR; whiskers extend 1.5 IQR beyond the hinges. A vertical red dashed line at $x = 0$ denotes neutral stance for reference.

Figure 5: Distribution of **Cultural Liberalism** Expressions by field

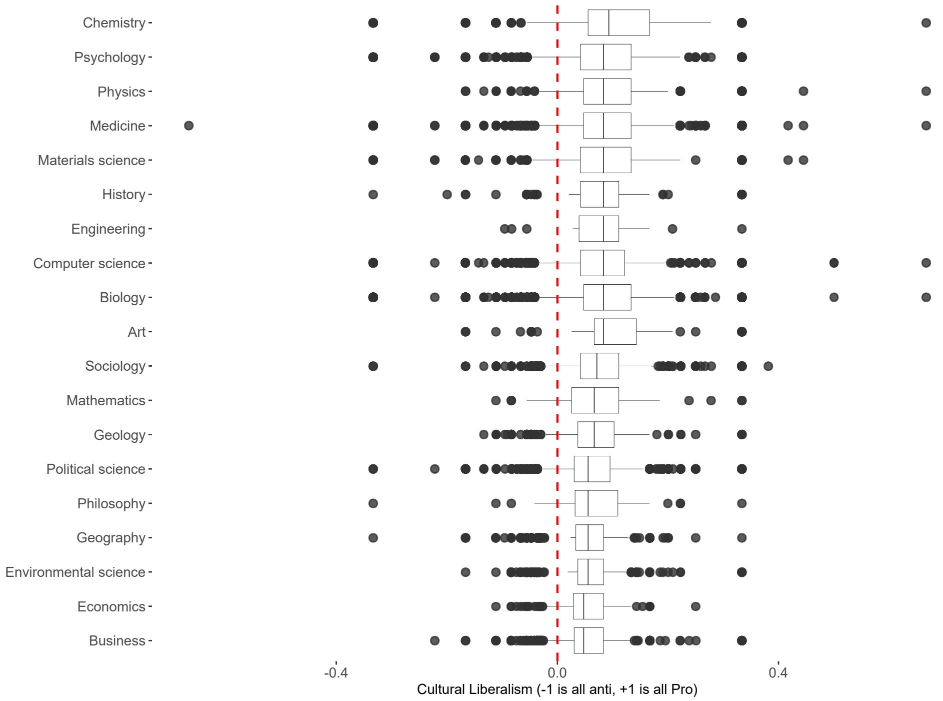

Data come from $\mathbf{n} = \mathbf{99{,}274}$ academics in a balanced sample (2016–2022), but here we focus on those with a *non-zero* ($\neq 0$) **Cultural Liberalism** stance (i.e. not neutral), with resulting sample size of $\mathbf{n} = \mathbf{6{,}074}$ academics. Zero-stances are excluded to emphasize distributions among authors who expressed at least some positive or negative position. Each box plot shows the distribution of per-academic mean stance (ranging from -1 = fully anti to +1 = fully pro), grouped by *field* and ordered in descending median stance. Center lines mark the median; boxes represent the IQR; whiskers extend 1.5 IQR beyond the hinges. A vertical red dashed line at $x = 0$ denotes neutral stance for reference.

Figure 6: Distribution of **Economic Collectivism** Expressions by field

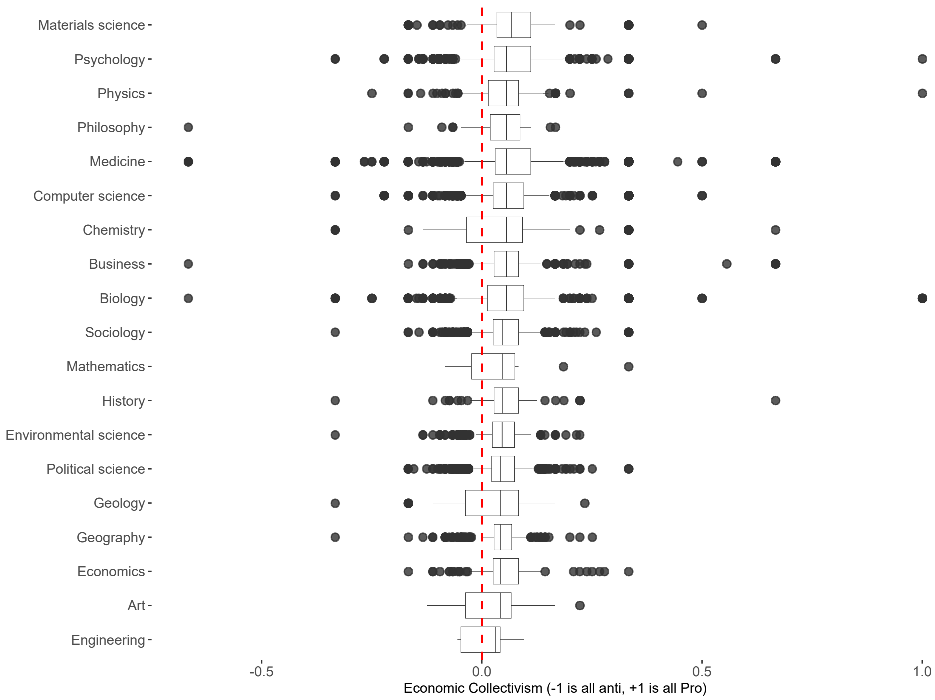

Data come from $\mathbf{n} = \mathbf{99{,}274}$ academics in a balanced sample (2016–2022), but here we focus on those with a *non-zero* ($\neq 0$) **Economic Collectivism** stance (i.e. not neutral), with resulting sample size of $\mathbf{n} = \mathbf{8{,}156}$ academics. Zero-stances are excluded to emphasize distributions among authors who expressed at least some positive or negative position. Each box plot shows the distribution of per-academic mean stance (ranging from -1 = fully anti to +1 = fully pro), grouped by *field* and ordered in descending median stance. Center lines mark the median; boxes represent the IQR; whiskers extend 1.5 IQR beyond the hinges. A vertical red dashed line at $x = 0$ denotes neutral stance for reference.

Figure 7: Correlates of Egocentric Expression

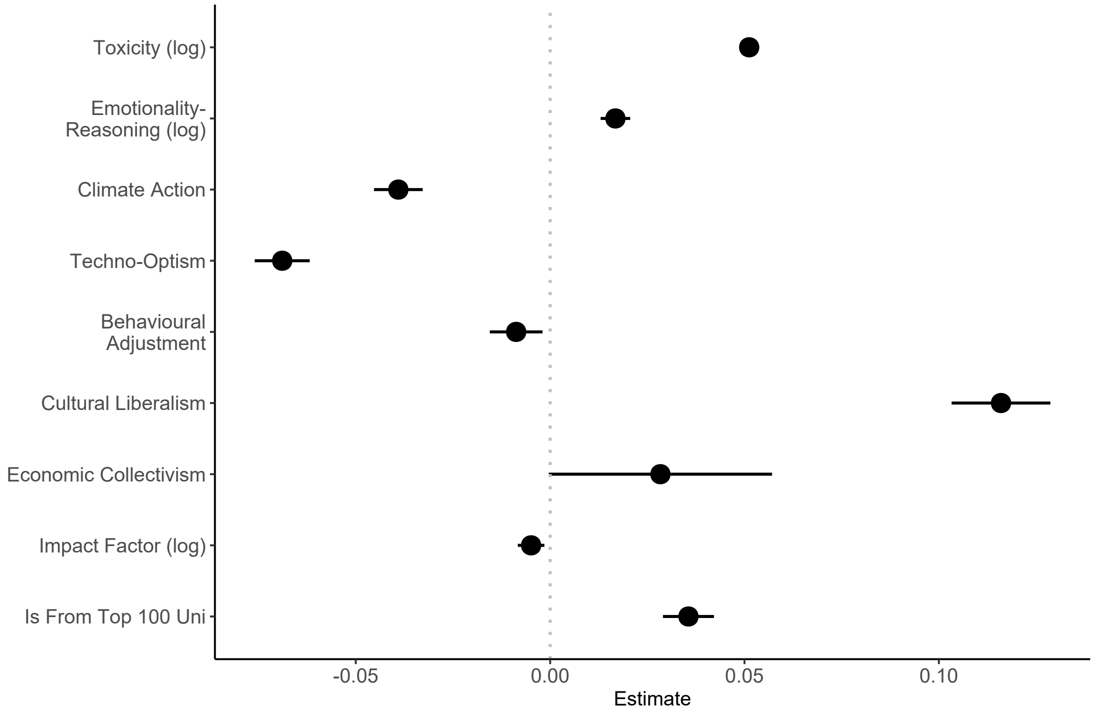

This figure reports Ordinary Least Squares (OLS) coefficient estimates (points) with 95% confidence intervals (horizontal lines) from fixed-effects regressions explaining *Egocentrism* (average first-person-singular words per tweet). Regressors include log-transformed *Toxicity*, log-transformed *Emotionality*, *Climate Action Stance*, *Techno-Optimism*, *Behavioral Adjustment*, *Cultural Liberalism*, *Economic Collectivism*, log-transformed *Impact Factor*, and a binary indicator for affiliation with a top-100 university. The dataset comprises a balanced panel of $\mathbf{n} = \mathbf{99{,}274}$ academics, observed via English tweets from 2016–2022. Each regression includes year-month fixed effects (to account for temporal trends), and standard errors clustered by author to address within-author correlations. All coefficients are point estimates, with 95% confidence intervals derived using cluster-robust standard errors. The vertical dotted line at zero marks the null hypothesis of no effect.

Figure 8: Correlates of Political Expression

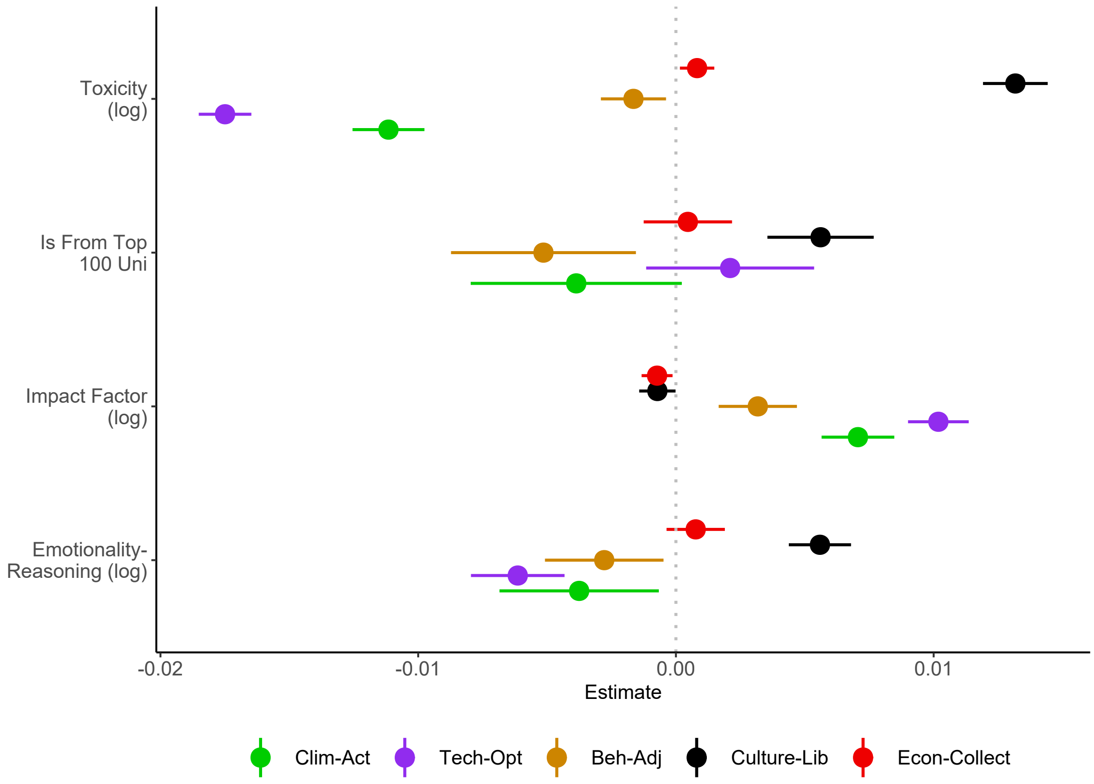

This figure presents regression coefficients (points) with 95% confidence intervals (lines) from separate fixed-effects models examining various political outcomes: *Climate Action*, *Techno-Optimism*, *Behavioral Adjustment*, *Cultural Liberalism*, and *Economic Collectivism*. Variables on the x-axis are log(*Toxicity*), log(*Emotionality*), log(*Impact Factor*), or an indicator variable "Is Top 100 University," depending on the model. Each model uses a panel dataset of $\mathbf{n} \approx \mathbf{99{,}274}$ academics, observed 2016–2022. Each regression includes year-month fixed effects (to capture time trends), and standard errors clustered by author to account for within-author correlations. All coefficients are point estimates (the center), with 95% CIs computed via cluster-robust standard errors (no multiple-comparison adjustments). The vertical dotted line at zero marks the null hypothesis of no effect.

Figure 9: Group Comparisons of Climate-Related Stances Controlling for Different Expressions

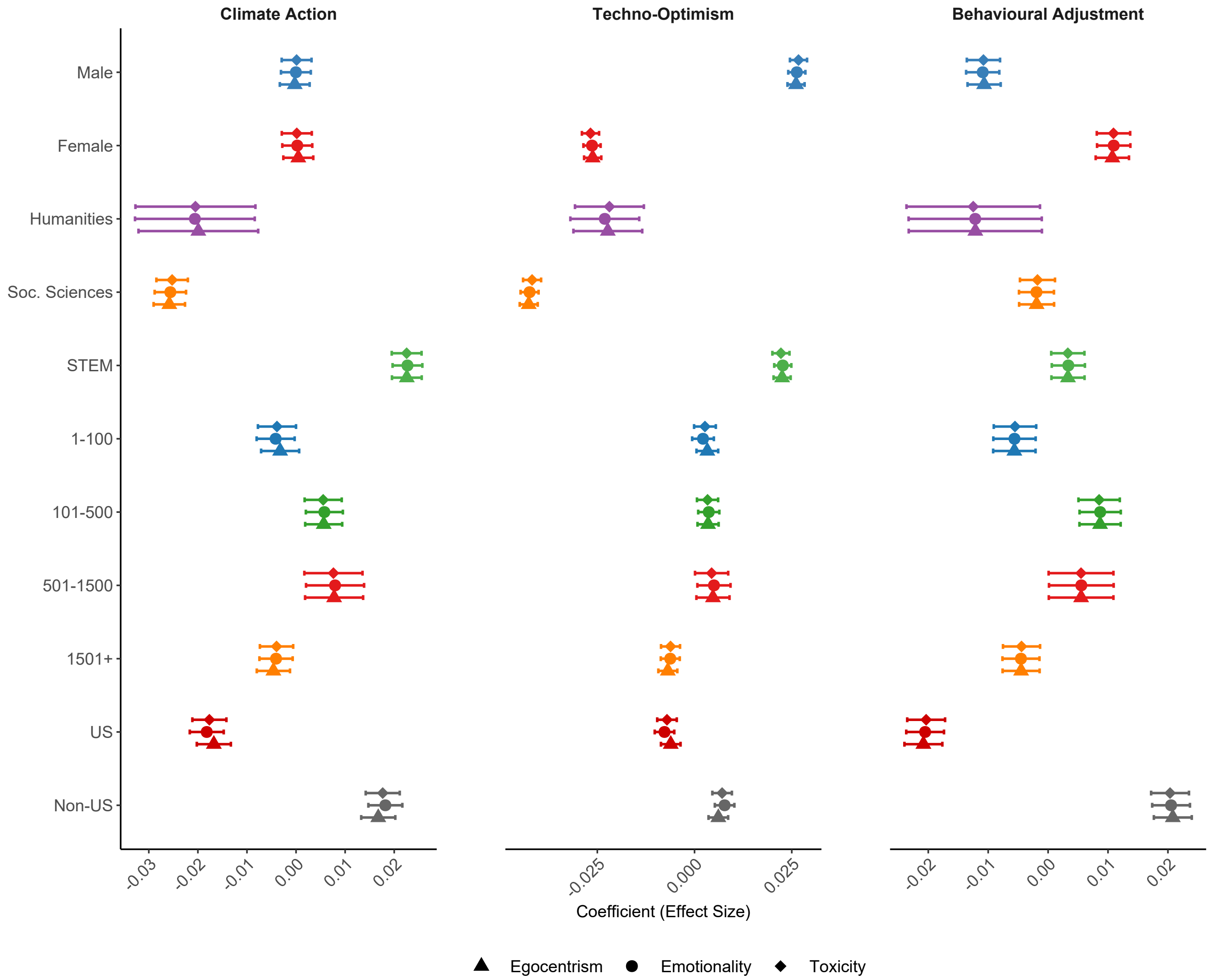

This figure presents coefficient estimates from linear regressions of three climate-related stances—**Climate Action**, **Techno-Optimism**, and **Behavioural Adjustment**—on subgroup indicators (e.g., Gender, Field, Country, and University Rankings) while controlling for one of three expression variables: **Emotionality**, **Egocentrism**, or **Toxicity**. Each point represents a regression coefficient, with error bars indicating ±95% confidence intervals derived from standard errors clustered by academic ($n = 151,792$, author-month observations). For each stance, three separate regressions were performed, each controlling for one expression variable (Emotionality, Egocentrism, or Toxicity). Statistical tests are two-sided, and no adjustments for multiple comparisons were made. Error bars denote confidence intervals computed via robust clustered standard errors. The center of each bar represents the estimated coefficient, and the horizontal dotted line at zero indicates no association. Results suggest that subgroup differences in stances are robust to controls for the "how" of expression style.

Figure 10: Group Comparisons of Economic and Cultural Stances Controlling for Different Expressions

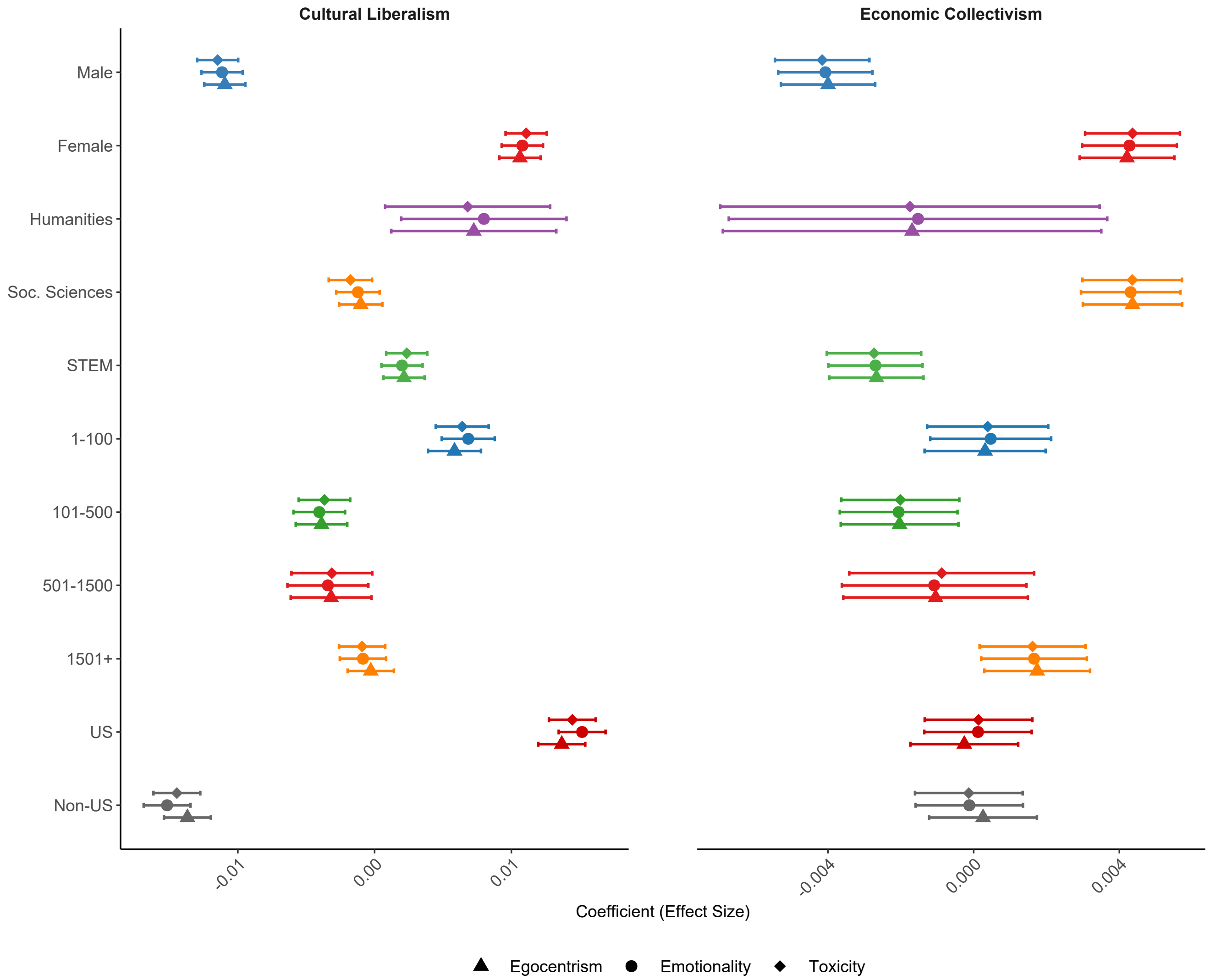

This figure presents coefficient estimates from linear regressions of two socio-political stances—**Cultural Liberalism** and **Economic Collectivism**—on subgroup indicators (e.g., Gender, Field, Country, and University Rankings) while controlling for one of three expression variables: **Emotionality**, **Egocentrism**, or **Toxicity**. Each point represents a regression coefficient, with error bars indicating ±95% confidence intervals derived from standard errors clustered by academic ($n = 151,792$, author-month observations). For each stance, three separate regressions were performed, each controlling for one expression variable (Emotionality, Egocentrism, or Toxicity). Statistical tests are two-sided, and no adjustments for multiple comparisons were made. Vertical error bars denote confidence intervals computed via robust clustered standard errors. The center of each bar represents the estimated coefficient, and the horizontal dotted line at zero indicates no association. Results suggest that subgroup differences in stances are robust to controls for the "how" of expression style.

Figure 11: Academic Expression Over Time by Gender

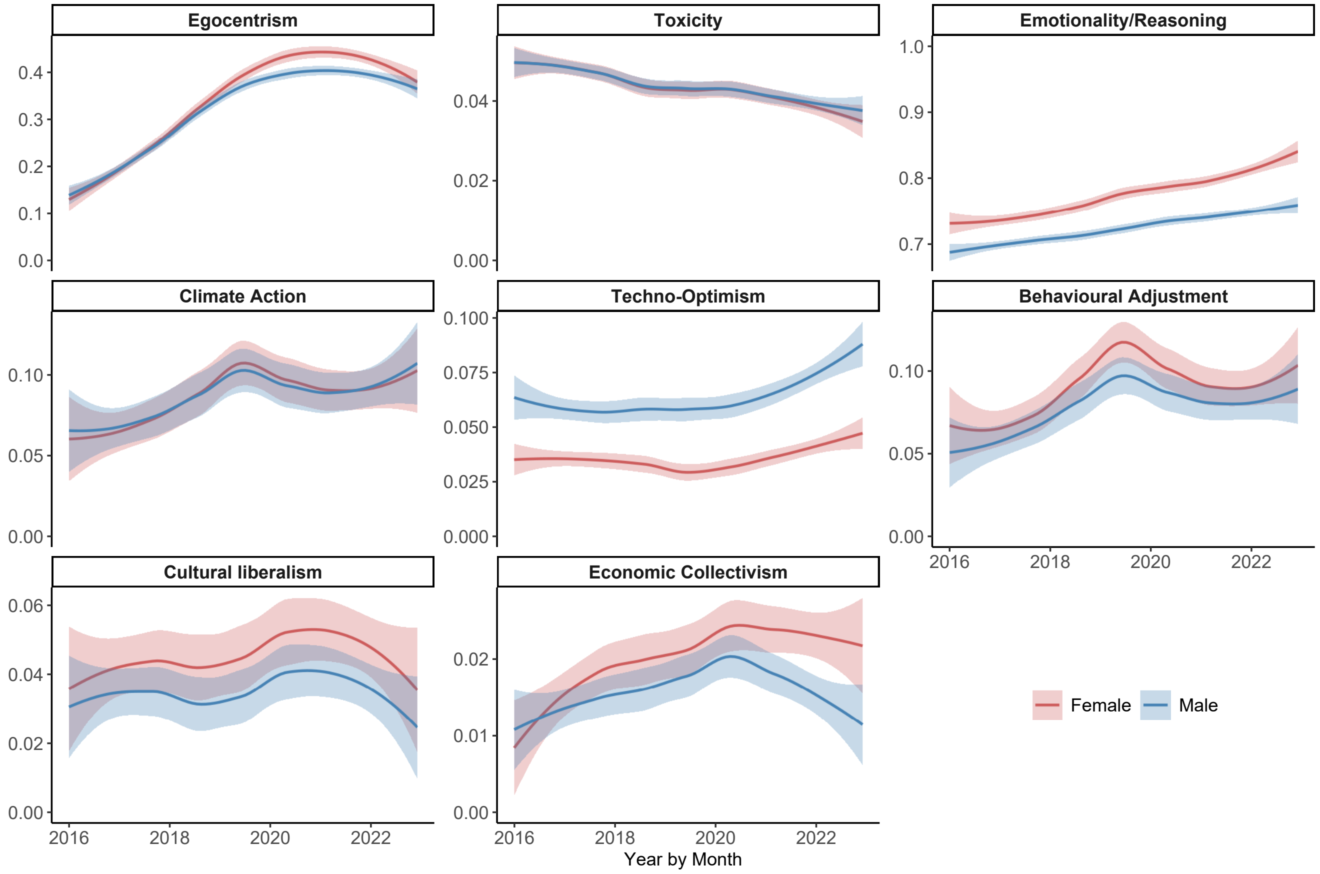

This figure presents the temporal dynamics of academic expression from January 2016 to December 2022, segmented by gender (**n** = **99,274** academics, balanced sample). Panels show distributions, smoothed trends, and variability across key behavioral and political metrics: **Egocentrism**, **Toxicity**, **Emotionality/Reasoning**, **Climate Action**, **Techno-Optimism**, **Behavioral Adjustment**, **Cultural Liberalism**, and **Economic Collectivism**. Each data point represents the monthly average for male and female academics, smoothed via LOESS with 95% confidence intervals around the trend line (shaded regions).

Figure 12: Academic Expression Over Time by Fields

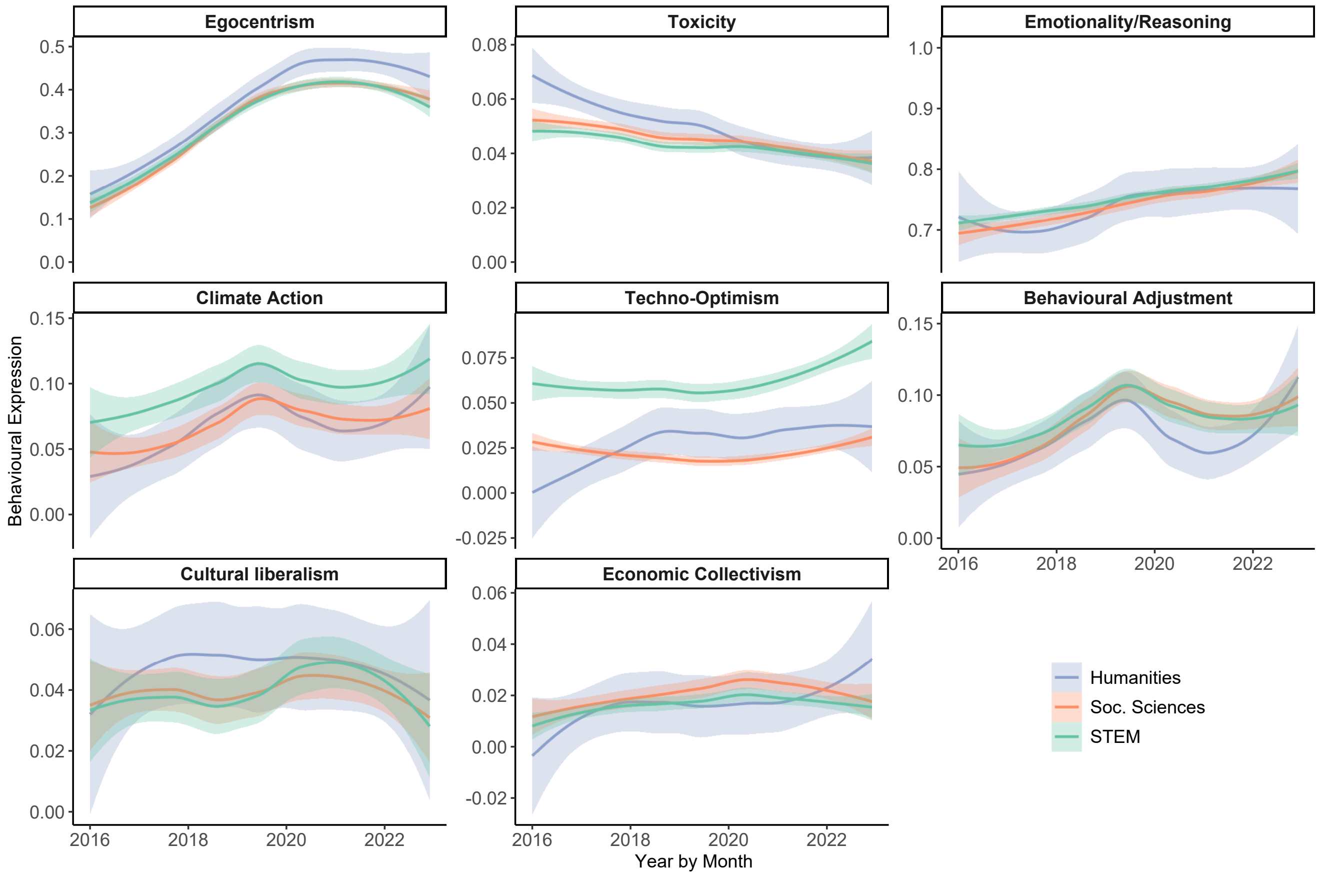

This figure presents the temporal dynamics of academic expression from January 2016 to December 2022, segmented by academic fields—Humanities, STEM, and Social Sciences (**n** = **99,274** academics, balanced sample). Panels show distributions, smoothed trends, and variability across key behavioral and political metrics: **Egocentrism**, **Toxicity**, **Emotionality/Reasoning**, **Climate Action**, **Techno-Optimism**, **Behavioral Adjustment**, **Cultural Liberalism**, and **Economic Collectivism**. Each data point represents the monthly average for each field, smoothed via LOESS with 95% confidence intervals around the trend line (shaded regions).

Figure 13: Academic Expression Over Time by Country (US vs. Non-US)

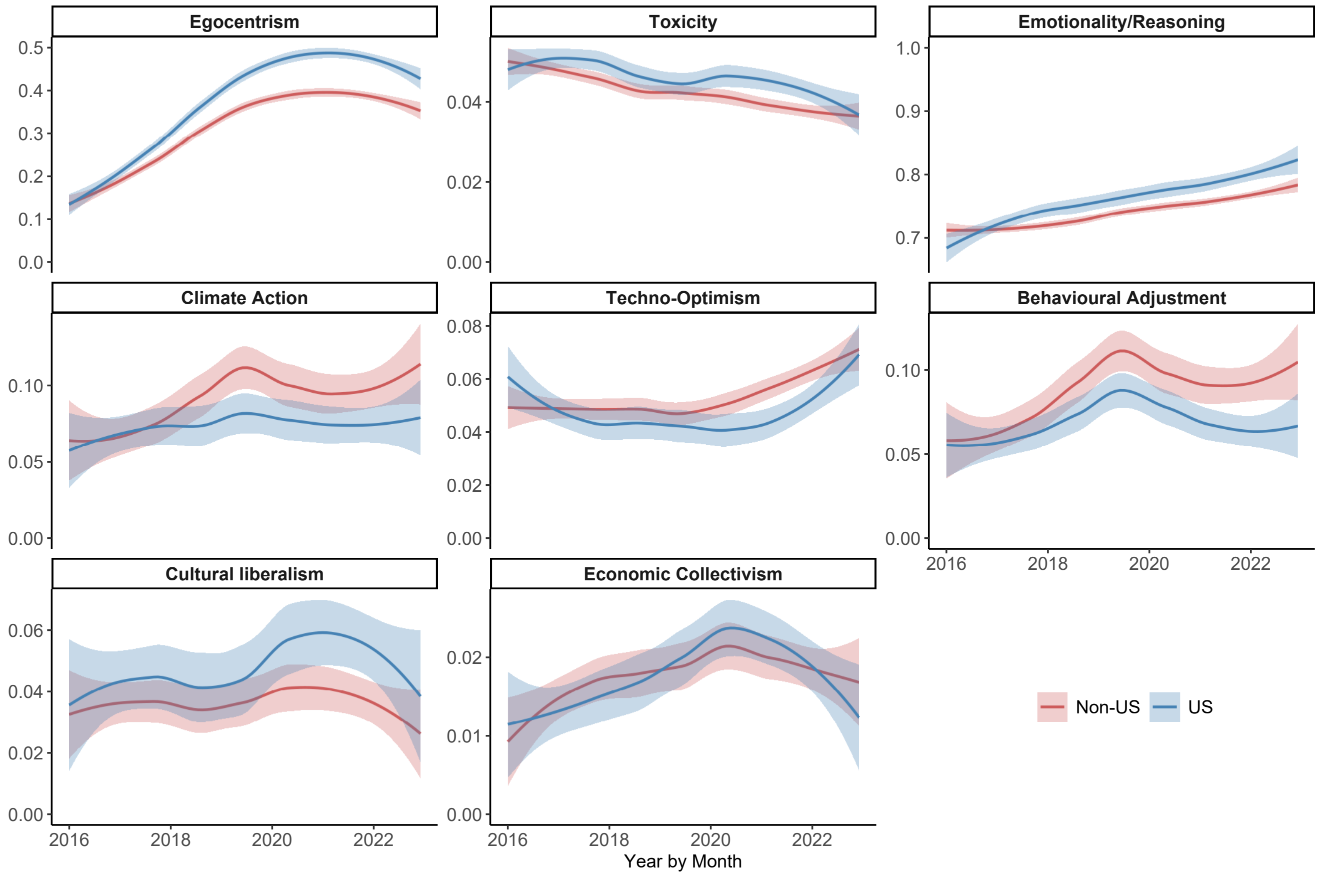

This figure presents the temporal dynamics of academic expression from January 2016 to December 2022, segmented by country of affiliation (US vs. Non-US; **n** = **99**,**274** academics, balanced sample). Panels show distributions, smoothed trends, and variability across key behavioral and political metrics: **Egocentrism**, **Toxicity**, **Emotionality/Reasoning**, **Climate Action**, **Techno-Optimism**, **Behavioral Adjustment**, **Cultural Liberalism**, and **Economic Collectivism**. Each data point represents the monthly average for US-based and non-US-based academics, smoothed via LOESS with 95% confidence intervals around the trend line (shaded regions).

Figure 14: Academic Expression Over Time by Twitter Reach vs. Academic Credibility

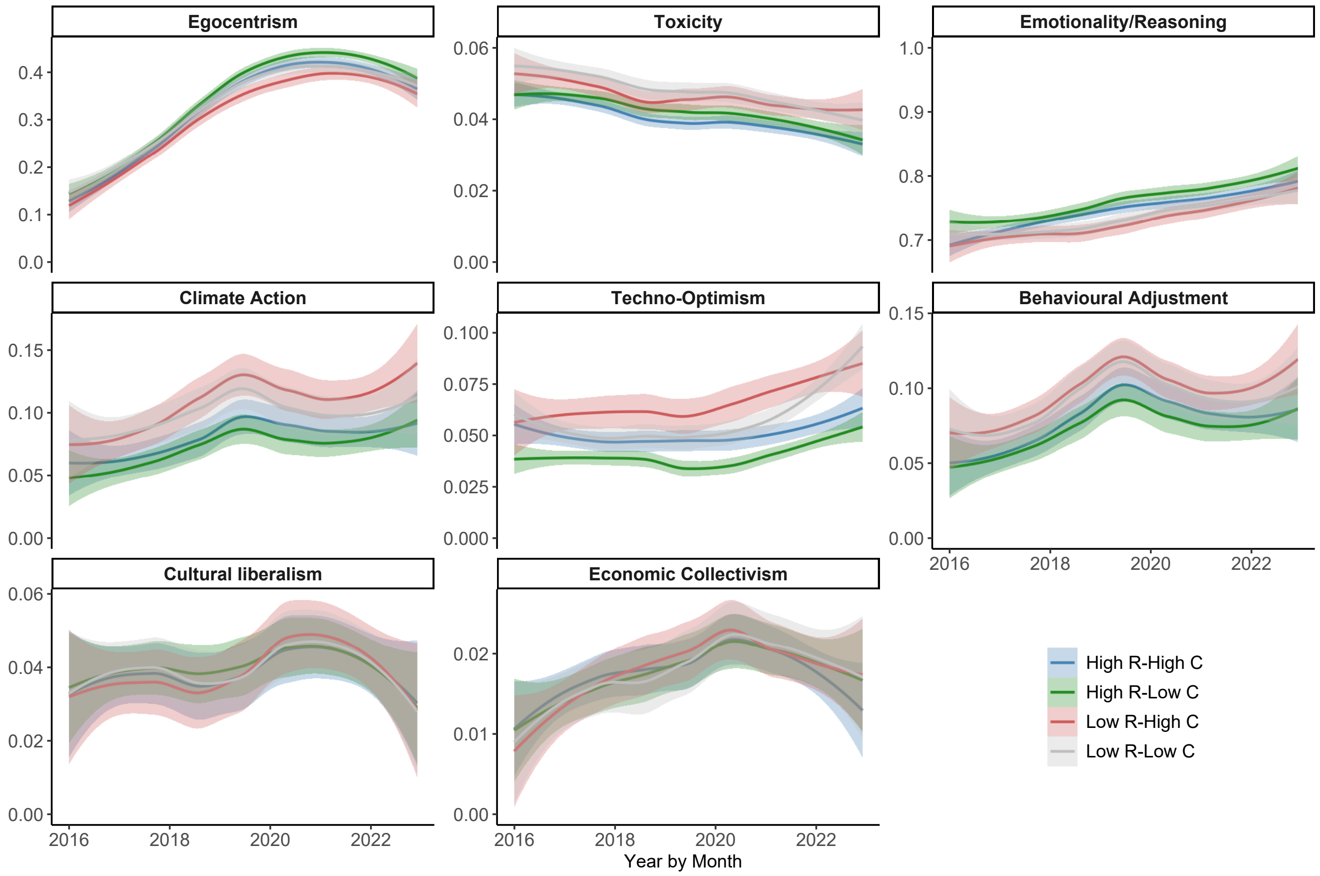

This figure presents the temporal dynamics of academic expression from January 2016 to December 2022, segmented by Twitter Reach (high vs. low) and Academic Credibility (high vs. low; **n = 99,274** academics, balanced sample). Panels show distributions, smoothed trends, and variability across key behavioral and political metrics: **Egocentrism**, **Toxicity**, **Emotionality/Reasoning**, **Climate Action**, **Techno-Optimism**, **Behavioral Adjustment**, **Cultural Liberalism**, and **Economic Collectivism**. For tone/style metrics (*how* academics communicate), credibility is based on whether an academic's publication impact factor is above or below the median within their field. For stance metrics (*what* academics express), credibility reflects whether an academic is a subject-matter expert on the topic under study. Twitter Reach is defined as the number of followers, categorized as high (above median) or low (below median). See Methods for detailed definitions and classification criteria. Each data point represents the monthly average for each subgroup, smoothed via LOESS with 95% confidence intervals around the trend line (shaded regions).

Figure 15: Average Number of Topical Tweets Over Time (2016–2022)

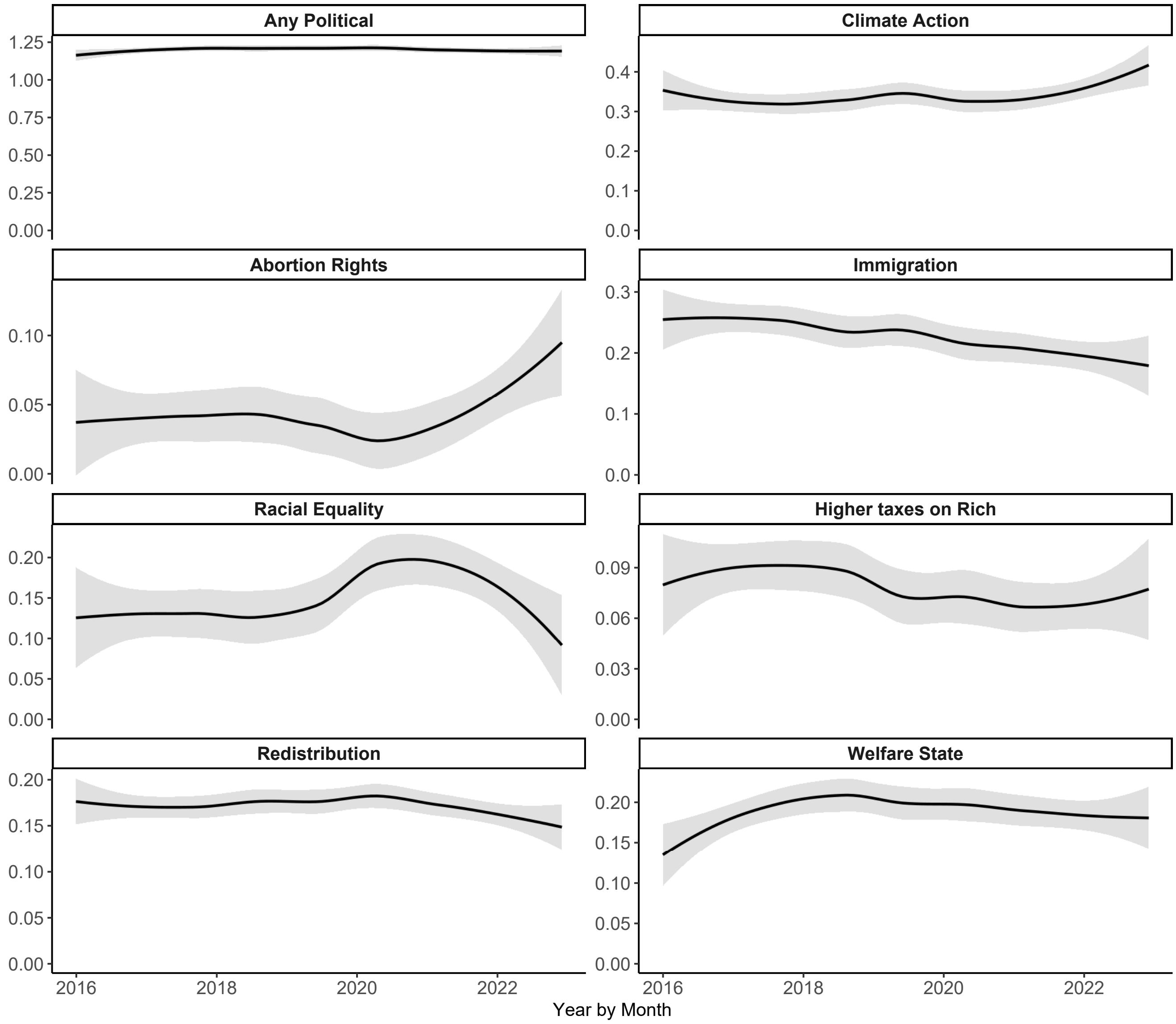

This figure presents the temporal dynamics of the average number of tweets per author per month across eight key topics: **Any Political**, **Climate Action**, **Abortion Rights**, **Immigration**, **Racial Equality**, **Higher Taxes on the Rich**, **Redistribution**, and **Welfare State**. The category "Any Political" aggregates all tweets expressing a stance (pro-, anti-, or neutral) across the remaining seven topics. Averages are computed at the author-month level. Trends are smoothed using LOESS with 95% confidence intervals (shaded regions). Results are derived from a balanced sample of $\mathbf{n} = \mathbf{99{,}274}$ academics who tweeted at least once early (January–June 2016) and late (July–December 2022) in the dataset. Refer to Methods for details on topic and stance classification, and see Table 7 for summary statistics.

Figure 16: Total Counts of Topical Tweets Over Time (2016–2022)

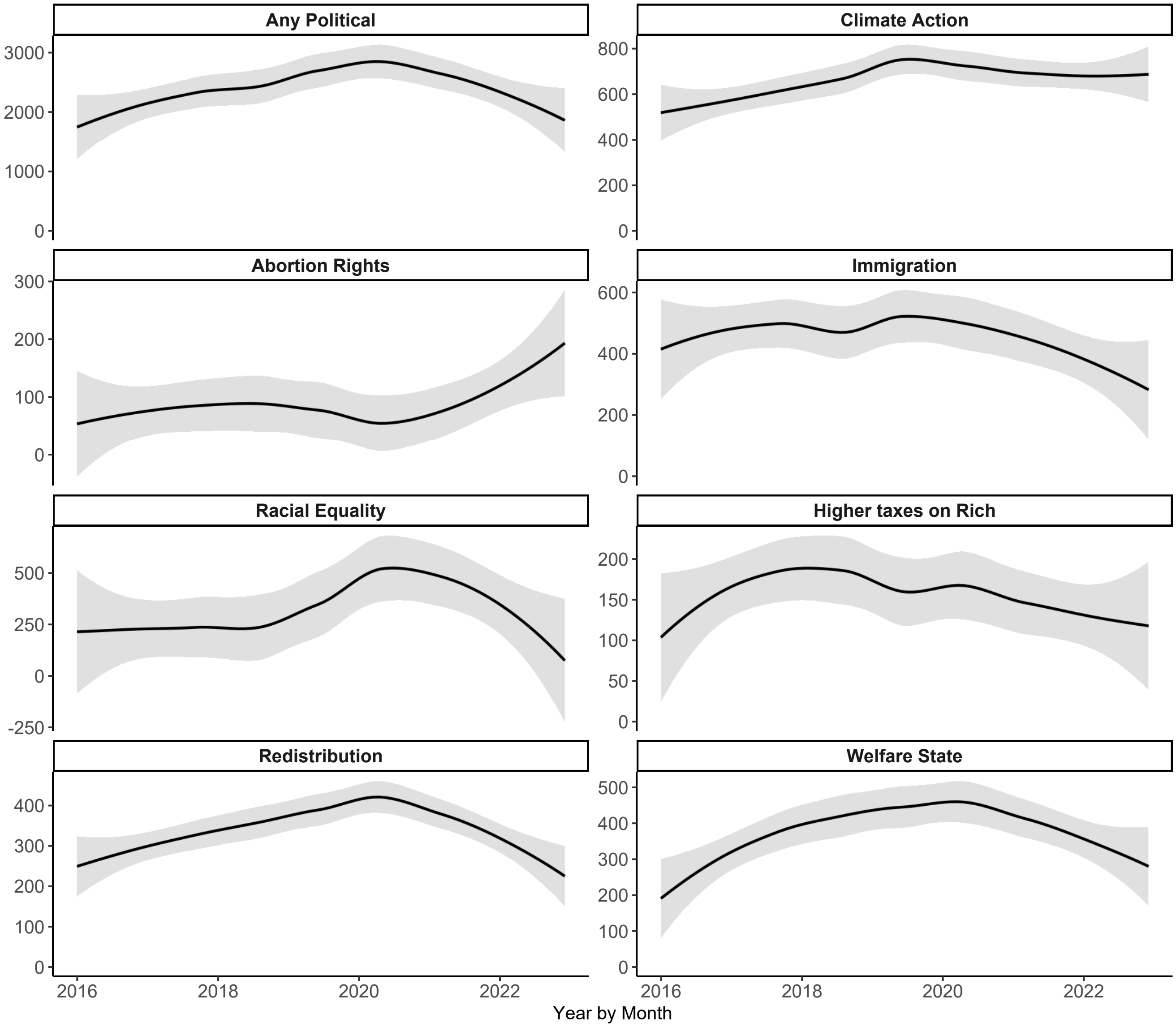

This figure presents the temporal dynamics of the absolute number of tweets per month across eight key topics: **Any Political**, **Climate Action**, **Abortion Rights**, **Immigration**, **Racial Equality**, **Higher Taxes on the Rich**, **Redistribution**, and **Welfare State**. The category "Any Political" aggregates all tweets expressing a stance (pro-, anti-, or neutral) across the remaining seven topics. Counts are computed monthly and smoothed using LOESS with 95% confidence intervals (shaded regions). Results are based on a balanced sample of $\mathbf{n} = \mathbf{99{,}274}$ academics who tweeted at least once early (January–June 2016) and late (July–December 2022) in the dataset. Refer to Methods for details on topic and stance classification, and see Table 7 for summary statistics.

Figure 17: Trends in Subcomponents of Cultural Liberalism and Economic Collectivism

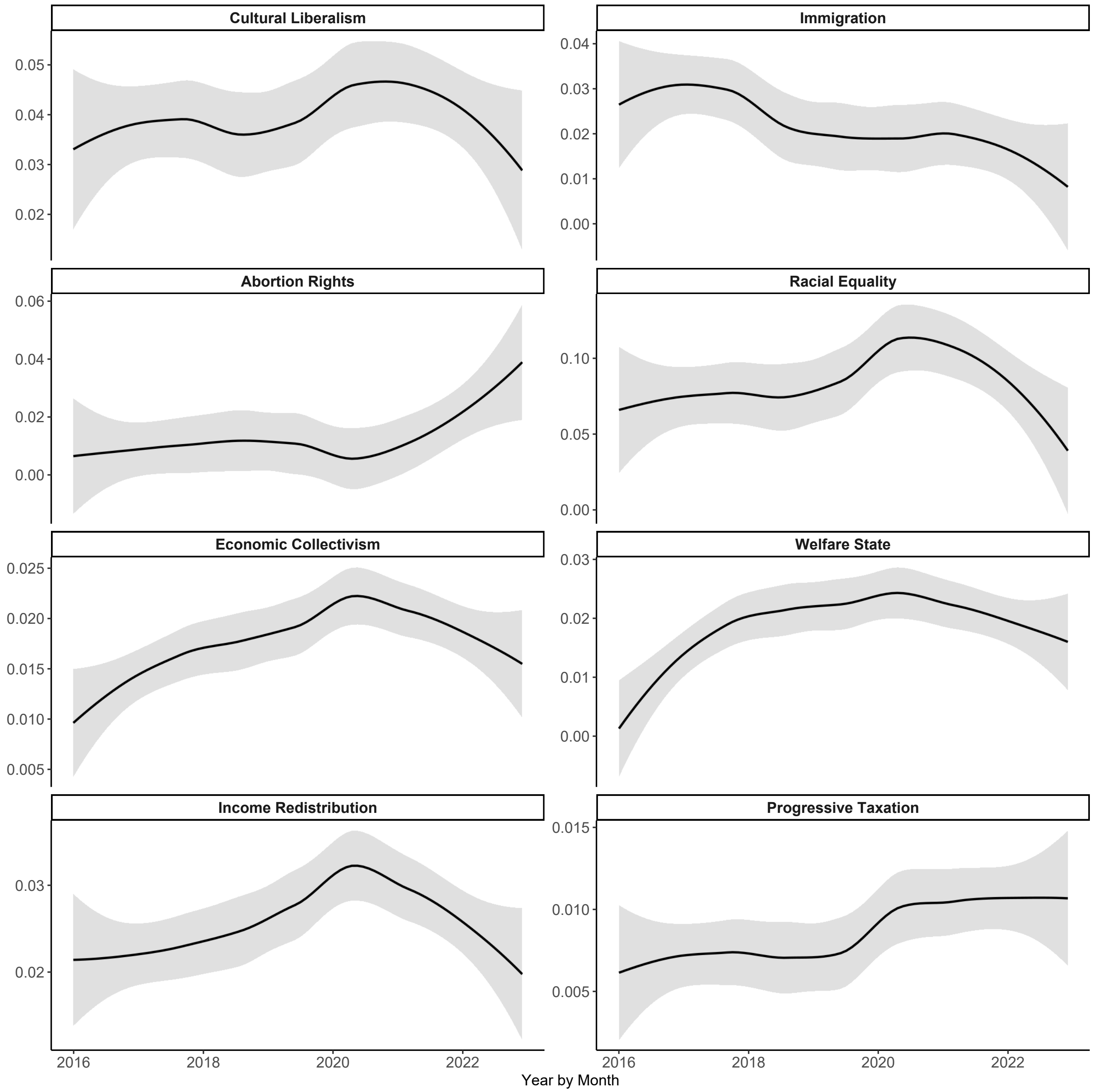

This figure presents the temporal dynamics of subcomponents within **Cultural Liberalism** and **Economic Collectivism** from January 2016 to December 2022. Subcomponents of Cultural Liberalism include **Abortion Rights**, **Immigration**, and **Racial Equality**, while those of Economic Collectivism include **Welfare State**, **Income Redistribution**, and **Progressive Taxation**. Each panel represents a distinct subtopic, with data points showing monthly averages across a balanced sample of $\mathbf{n} = \mathbf{99{,}274}$ academics. Trends are smoothed using LOESS with 95% confidence intervals (shaded regions).

Figure 18: Within-User Trends in Academic Expression Over Time (Main Sample)

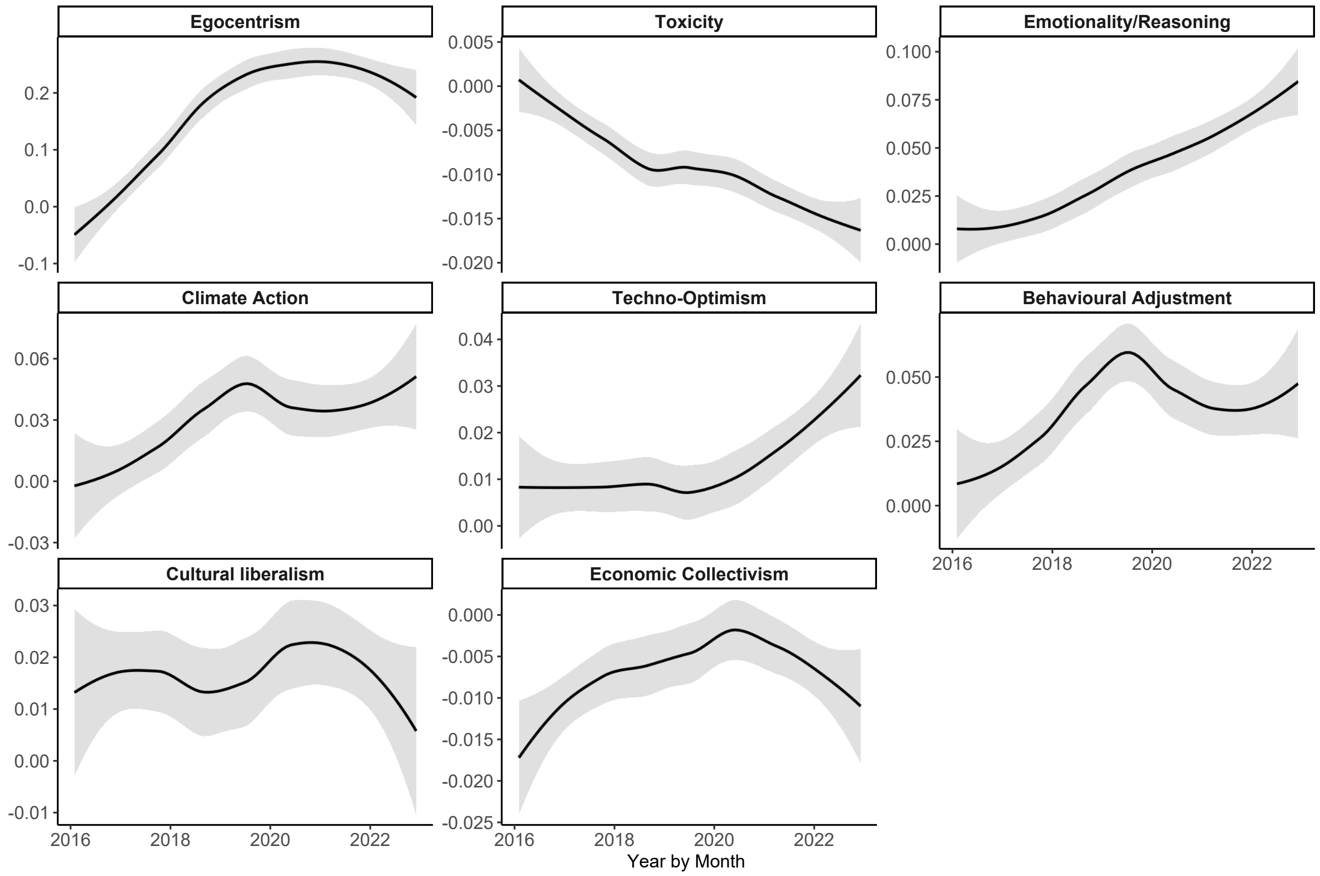

This figure presents within-user temporal dynamics of academic expression from January 2016 to December 2022, using a balanced sample of **n = 99,274** academics who tweeted at least once in both the early (January–June 2016) and late (July–December 2022) periods of the dataset. Trends are adjusted for individual fixed effects, isolating temporal changes within users while controlling for persistent individual characteristics. Panels show adjusted trends across behavioral and political metrics, smoothed using LOESS with 95% confidence intervals (shaded regions).

Figure 19: Within-User Trends in Academic Expression by Gender

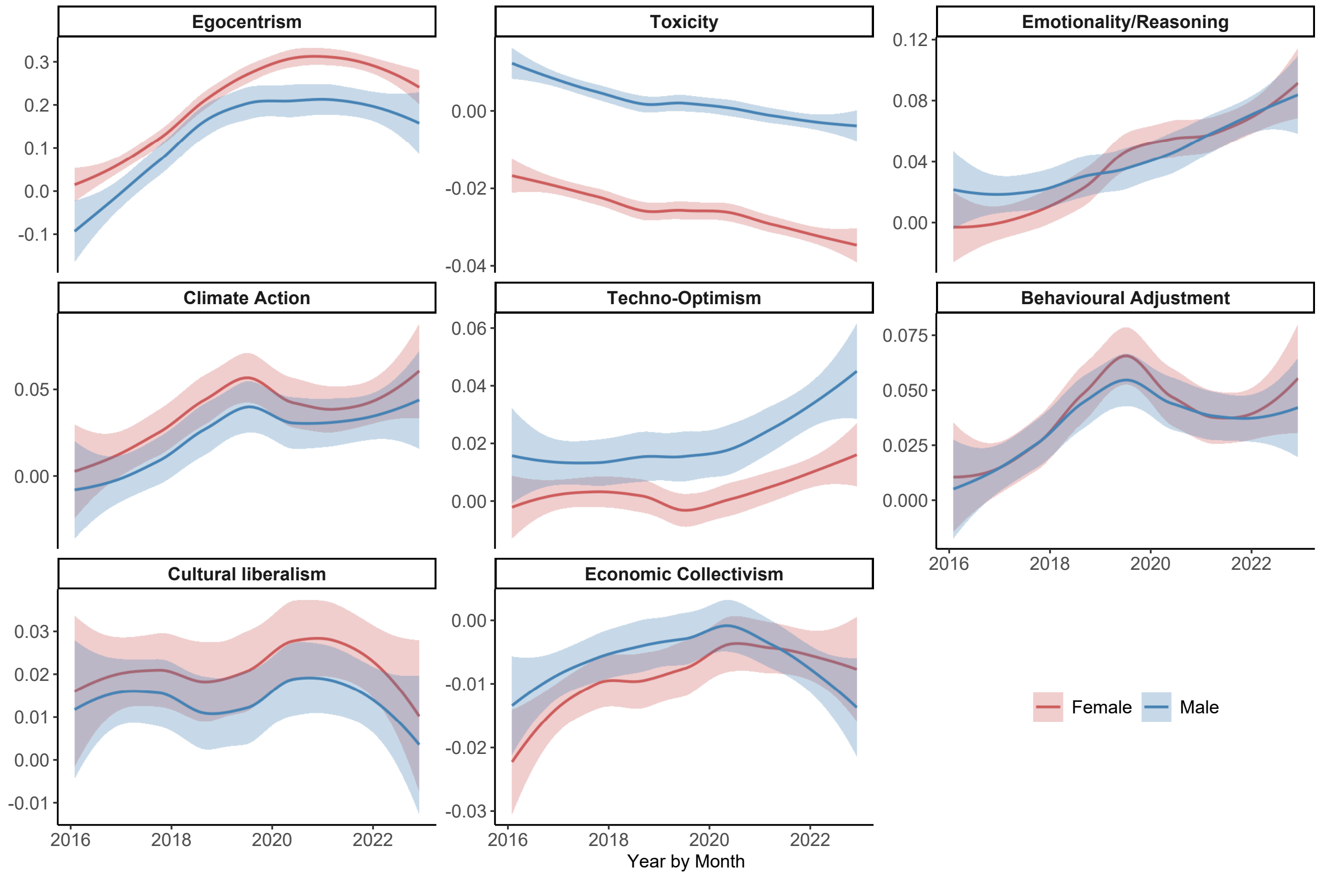

This figure presents within-user temporal dynamics of academic expression from January 2016 to December 2022, segmented by gender (**Male**, **Female**), using a balanced sample of **n** = **99**,**274** academics. Trends are adjusted for individual fixed effects, allowing for the isolation of temporal changes within users while controlling for persistent individual characteristics. Panels show adjusted trends across behavioral and political metrics, smoothed using LOESS with 95% confidence intervals (shaded regions).

Figure 20: Within-User Trends in Academic Expression by Field of Study

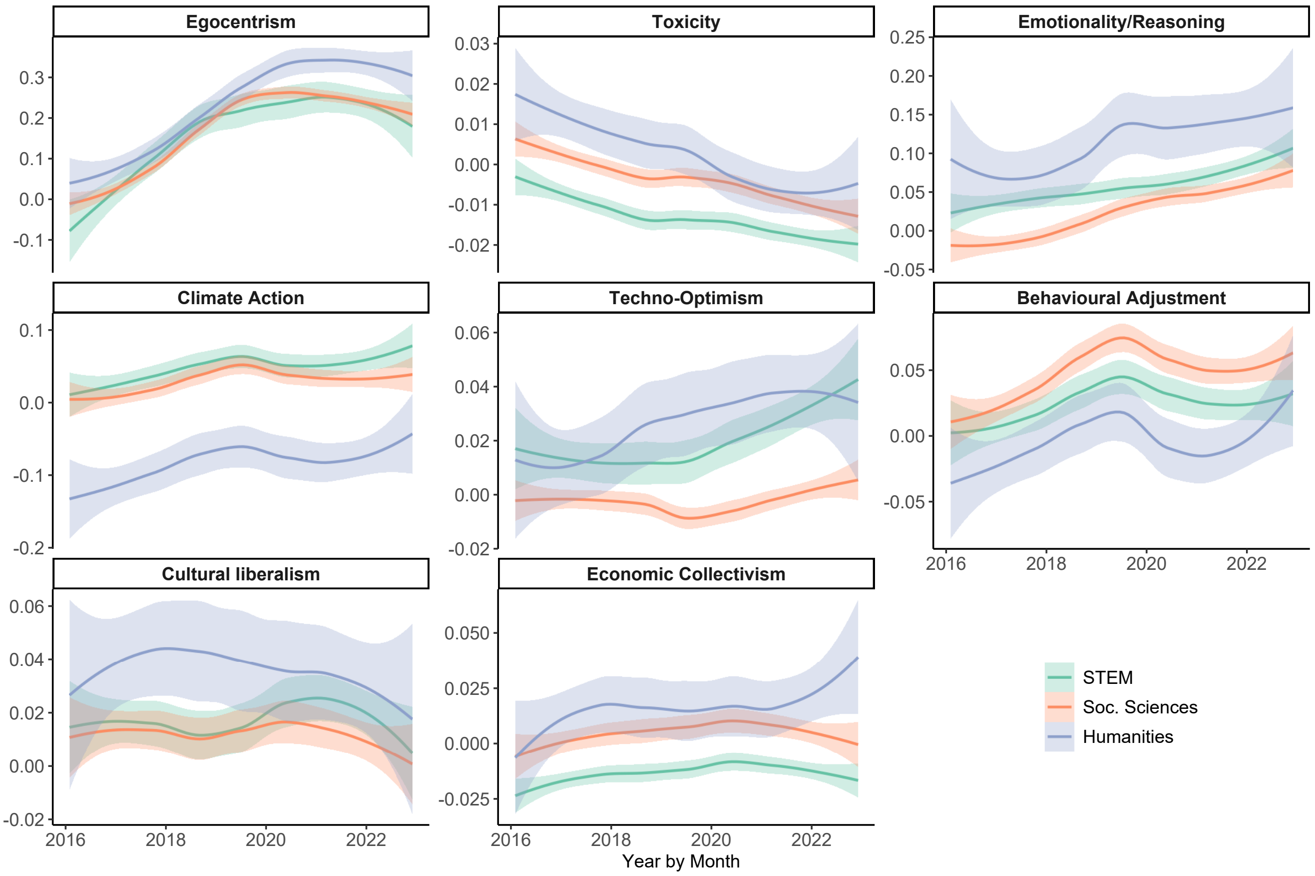

This figure presents within-user temporal dynamics of academic expression from January 2016 to December 2022, segmented by field of study (**STEM**, **Social Sciences**, **Humanities**), using a balanced sample of **n** = **99**,**274** academics. Trends are adjusted for individual fixed effects, isolating temporal changes within users while controlling for persistent individual characteristics. Panels show adjusted trends across behavioral and political metrics, smoothed using LOESS with 95% confidence intervals (shaded regions).

Figure 21: Within-User Trends in Academic Expression by Country of Institution

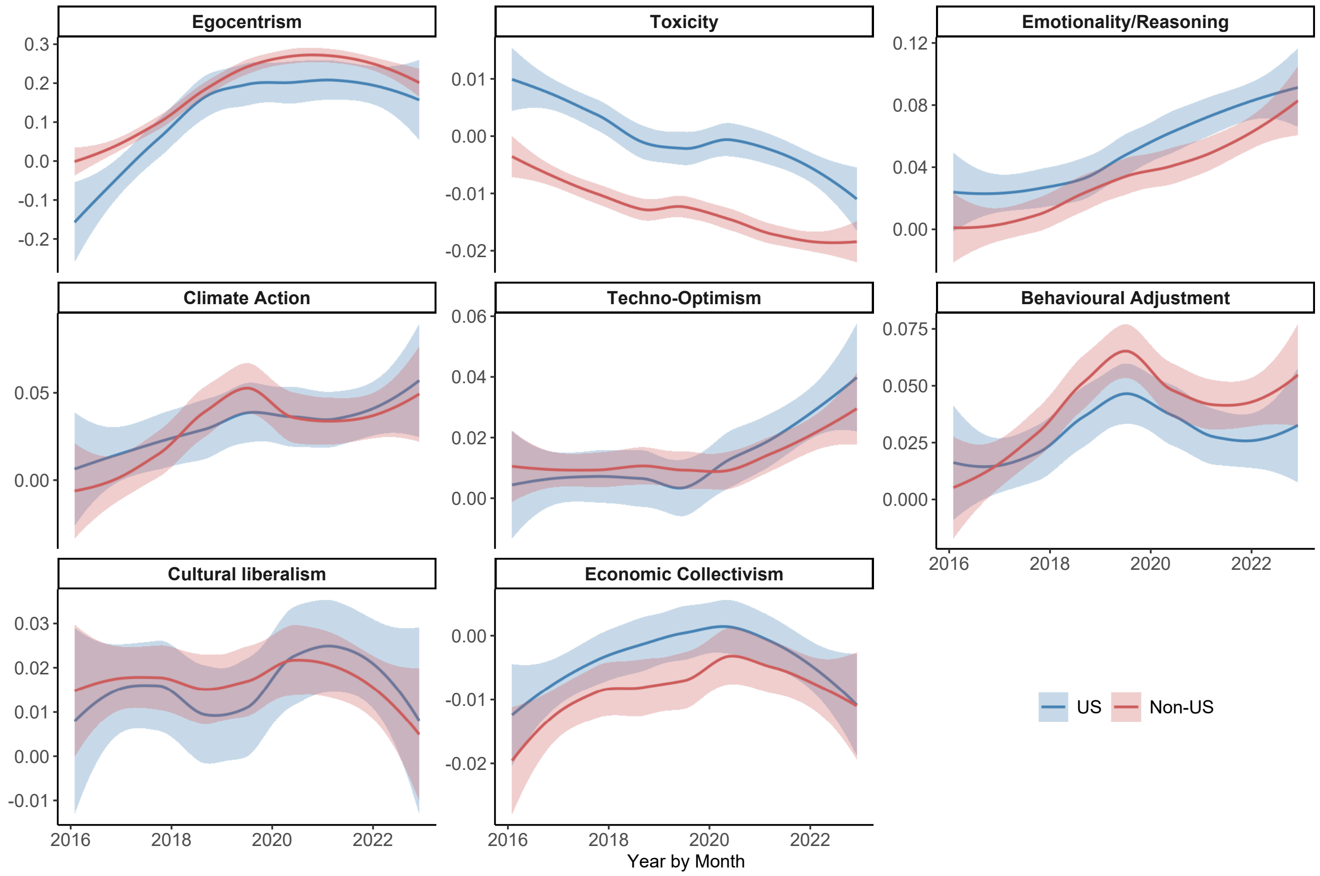

This figure presents within-user temporal dynamics of academic expression from January 2016 to December 2022, segmented by the geographical location of academics (**US-based**, **Non-US-based**), using a balanced sample of $\mathbf{n} = \mathbf{99{,}274}$ academics. Trends are adjusted for individual fixed effects, allowing for the isolation of temporal changes within users while controlling for persistent individual characteristics. Panels show adjusted trends across behavioral and political metrics, smoothed using LOESS with 95% confidence intervals (shaded regions).

Table 10: Random Sample of Terms for Topics Detected using GPT-4

Topic	Terms
Abortion Rights	roe v. wade 2021 debate, #abortionaccess, #abortionlaw, my body my, #prolife, abortion restriction laws, stop the bans, justice for abortion rights, georgia abortion ban, repeal hyde amendment, abortion access in 2017, #stopthebans, partial birth, texas abortion bill, telemedicine for abortion, abortion, abortion ban laws, #standwithpp, gestational age limits, pro life anti abortion
Climate Action	#netzero, climate change and agriculture, climate change mitigation, united nations climate, carbon footprint, climate change 2013, earth temperatures, protect our planet, scientific consensus on climate, global climate change, climate change convention, stop global warming now, #cop21 paris, paris climate agreement 2015, greenhouse gas emission reduction, climate action summit 2019, carbon capture storage, trump climate change, climate change adaptation, global average temperature
Immigration	border security and enforcement, stricter immigration laws, bipartisan framework for comprehensive immigration, customs and border, scrapping the diversity lottery, #deportationforce, asylum seeking policies, travel ban supreme court, #refugeeasylum, secure border initiative, legal status pathway, us immigration policy, fight against deportation, ban on muslim countries, support immigration reform, family detention centers, 2017 sanctuary cities controversy, comprehensive immigration reform 2022, abolish ice movement, migrant detention
Racism or Race Relations	ethnic minorities, race-related hate crimes, reparations for african americans, #charlestonshooting, racism in sports 2008, stand against racism 2022, hate-crime, racial identity, #racismisavirus, racial inequality awareness, protests against racial injustice, black lives matter, racial inequality in 2007, blacklivesmatter, racial profiling in police, address racial disparities, racial equality now, color should not matter, #whitesupremacy, say no to racism
Redistribution of Income	wealth tax proposals, redistribution of income, taxation for redistribution, economic inequality issues, wealth redistribution policies, income redistribution efforts, redistribution debate, income redistribution policies, redistribution, progressive income redistribution, income gap widening, tax on wealth, #progressivetax for fairness, income redistribution and economy, progressive tax system, income redistribution mechanisms, economic redistribution strategies, basic income, #redistribution, top1percent
Tax Policy	cryptocurrency tax rules, #irs, digital tax policy, tax rate adjustments, obama tax policy, tax code simplification, tax cuts, #obamatax, tax bracket changes, small business tax, tax policy under obama, tax policy review, trump tax cuts, income tax rates, tax legislation, tax avoidance, impact of taxes, tax policy debates, #taxes, property tax
Welfare State	public housing assistance programs, welfare state critique, #socialsecurity, public pension scheme reform, welfare budget, income redistribution policy, social expend, public healthcare, social welfare policies, food stamps, welfare rights, welfare state issues, food stamp program 2013, reform the welfare state, benefit system, unemployment benefits policy 2018, welfare reform policies, economic impact welfare state, #publicwelfare, welfare state development

This table presents a random sample of terms identified for topics such as Abortion Rights, Climate Action, Immigration, and others, using GPT-4. The terms were generated through dynamic, context-aware dictionary creation, employing prompt engineering across multiple years (2016–2022) and varying ngram types (unigrams, bigrams, etc.). These terms encompass both policy-related discourse and Twitter-specific vernacular, reflecting the evolving nature of online conversations. For instance, *roe v. wade 2021 debate* and *#prolife* under "Abortion Rights," and *#netzero* under "Climate Action" highlight the adaptability of the model to identify key terms relevant to evolving public and policy discussions. The identified terms form the basis for subsequent stance detection and analysis. Refer to Methods for details on the dictionary creation process and its applications in detecting contextually significant terms over time.

Table 13: Evaluation Metrics for Topic Detection Validation

Topic	Precision	Recall	F1-Score
Legalization of Abortion	86.51	66.00	74.87
Hillary Clinton	75.49	60.79	67.35
Feminist Movement	51.00	97.67	67.01
Donald Trump	79.41	65.59	71.84
Overall	65.47	74.32	69.62

Table shows precision, recall, and F1-Score metrics for topic detection. The results highlight the effectiveness of the topic dictionaries generated using GPT-4 and the robustness of the methodology. Tweets were matched against the dictionaries, and true and false positives and negatives were calculated. For instance, a true positive (TP) occurs when a tweet labeled as "Legalization of Abortion" contains a term from the corresponding dictionary, while a true negative (TN) occurs when no terms from the dictionary match tweets labeled for other topics. The high recall demonstrates the comprehensive nature of the dictionaries, which capture the evolving lexicon over the years, while the precision shows the effectiveness of filtering out unrelated tweets. This approach ensures accurate topic detection across diverse tweet datasets by leveraging context-aware keyword dictionaries and refining selections to enhance accuracy.

Table 14: Evaluation Metrics for Gender Classification

Metric	Unweighted	Weighted by Count
Precision	0.8097	0.9866
Recall	0.8202	0.9871
F1 Score	0.8149	0.9868
Accuracy	0.8610	0.9863

Validation results for the gender classification method using a dataset of 147,269 unique names from authoritative sources, including US Social Security Card Applications (1880-2019), UK Baby Names (2011-2018), British Columbia's Baby Names (1918-2018), and Australia's Popular Baby Names (1944-2019). The GPT-3.5-turbo model classified names as Male, Female, or Unclear. The table presents both unweighted and weighted metrics: Precision, Recall, F1 Score, and Accuracy. Unweighted metrics show high overall performance, treating all names equally. Weighted metrics, which give more importance to frequently occurring names, yield even higher scores, particularly for names more likely expected to appear in our dataset. This demonstrates the method's strong accuracy and robustness, especially for common names.

Figure 22: Spatio-temporal correlation between Twitter and GSS stance on Climate Action

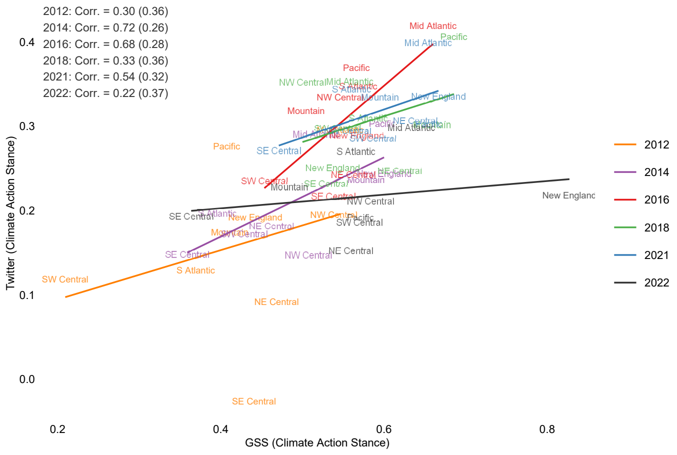

This figure presents the spatio-temporal relationship between regional net stances on Climate Action derived from Twitter and the General Social Survey (GSS) for the years 2012–2022. Each point represents a U.S. region-year observation, with colours distinguishing years. The x-axis represents Twitter stances aggregated by region and year, while the y-axis represents GSS stances for corresponding years and regions. A linear regression line with a 95% confidence band is fitted to examine the association. The correlation coefficient (r) and corresponding p-value, along with the sample size ($n = 54$, nine regions observed across six years), are annotated on the plot. Statistical tests are two-sided, and no adjustments for multiple comparisons were made. Methods discuss data sources, stance calculation, and potential biases in GSS metrics (e.g., environmental spending perceptions). The comparison highlights moderate correlations, emphasizing that while stances align regionally, methodological differences in measurement introduce noise.

Figure 23: Spatio-temporal correlation between Twitter and GSS stance on Abortion Rights

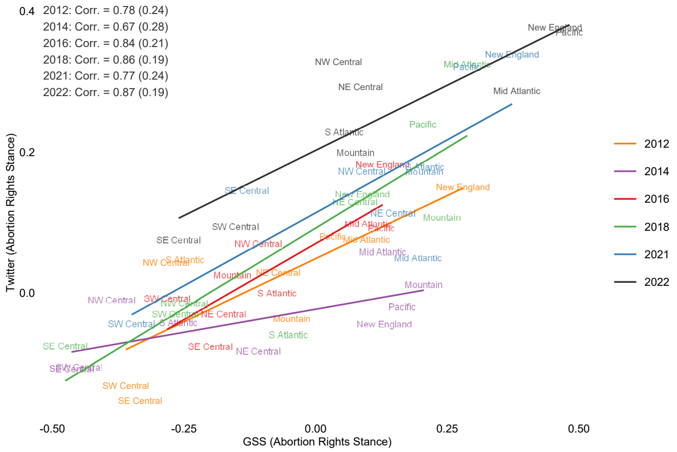

This figure illustrates the spatio-temporal correlation between regional net stances on Abortion Rights derived from Twitter and the General Social Survey (GSS) for 2012–2022. Each point represents a U.S. region-year observation, colour-coded by year. The x-axis shows Twitter stances aggregated by region and year, while the y-axis represents GSS stances for corresponding years and regions. A linear regression line with a 95% confidence band is fitted to the data. The correlation coefficient (r) and corresponding p-value, along with the sample size ($n = 54$, nine regions observed across six years), are reported on the plot. Statistical tests are two-sided, with no adjustments for multiple comparisons. The GSS stance on abortion rights ($GSS_u^{AbortionRights} = \frac{Yes-No}{Yes+No}$) focuses solely on respondents' social values, leading to stronger alignment with Twitter stances than topics with multidimensional GSS questions (e.g., Climate Action). Methods describe sampling, stance metrics, and cross-sectional alignment across the two datasets.

References

[1] Saif Mohammad, Svetlana Kiritchenko, Parinaz Sobhani, Xiaodan Zhu, and Colin Cherry. Semeval-2016 task 6: Detecting stance in tweets. In *Proceedings of the 10th international workshop on semantic evaluation (SemEval-2016)*, pages 31–41, 2016.

[2] Guido Zarrella and Amy Marsh. Mitre at semeval-2016 task 6: Transfer learning for stance detection. *arXiv preprint arXiv:1606.03784*, 2016.

[3] Aaron Clauset, Cosma Rohilla Shalizi, and Mark EJ Newman. Power-law distributions in empirical data. *SIAM review*, 51(4):661–703, 2009.

[4] James W Pennebaker, Ryan L Boyd, Kayla Jordan, and Kate Blackburn. The development and psychometric properties of liwc2015. Technical Report, University of Texas at Austin, 2015.
