# Supplementary material for: Political expression of academics on Twitter
Source: Nat Hum Behav. 2025 Jun 3;9(9):1815–32. doi: 10.1038/s41562-025-02199-1 (PMC12454119; doi:10.1038/s41562-025-02199-1)
Supplement: Supplementary file 2 — Reporting Summary [file 41562_2025_2199_MOESM2_ESM.pdf]

nature portfolio

Corresponding author(s): Prashant Garg

Last updated by author(s): Mar 31, 2025

Reporting Summary

Nature Portfolio wishes to improve the reproducibility of the work that we publish. This form provides structure for consistency and transparency in reporting. For further information on Nature Portfolio policies, see our Editorial Policies and the Editorial Policy Checklist.

Statistics

For all statistical analyses, confirm that the following items are present in the figure legend, table legend, main text, or Methods section.

n/a	Confirmed	
☐	☒	The exact sample size (*n*) for each experimental group/condition, given as a discrete number and unit of measurement
☒	☐	A statement on whether measurements were taken from distinct samples or whether the same sample was measured repeatedly
☐	☒	The statistical test(s) used AND whether they are one- or two-sided<br>*Only common tests should be described solely by name; describe more complex techniques in the Methods section.*
☐	☒	A description of all covariates tested
☐	☒	A description of any assumptions or corrections, such as tests of normality and adjustment for multiple comparisons
☐	☒	A full description of the statistical parameters including central tendency (e.g. means) or other basic estimates (e.g. regression coefficient) AND variation (e.g. standard deviation) or associated estimates of uncertainty (e.g. confidence intervals)
☐	☒	For null hypothesis testing, the test statistic (e.g. F, t, r) with confidence intervals, effect sizes, degrees of freedom and P value noted<br>*Give P values as exact values whenever suitable.*
☒	☐	For Bayesian analysis, information on the choice of priors and Markov chain Monte Carlo settings
☒	☐	For hierarchical and complex designs, identification of the appropriate level for tests and full reporting of outcomes
☐	☒	Estimates of effect sizes (e.g. Cohen's *d*, Pearson's *r*), indicating how they were calculated

Our web collection on statistics for biologists contains articles on many of the points above.

Software and code

Policy information about availability of computer code

Data collection	The data collection process for this study utilized several tools and software. We accessed Twitter's academic API using the R packages academictwitteR (version 0.2.3) and rtweet (version 0.7.0). The raw tweets were collected in January 2023 and included tweets from users who consented to the public availability of their data. To ensure compliance with Twitter's policies, only aggregate measures at the individual-by-time level are provided. The raw tweets are securely stored on Google's BigQuery SQL database and are accessible upon request, subject to Twitter's data-sharing policies. Additionally, data about academics were accessed through OpenAlex using the R package openalexR (version 0.1.1). The collection processes were conducted using R (version 4.3.0).
Data analysis	Data analysis was conducted using R (version 4.3.0) and Python (version 3.11.4). In R, the following packages were utilized: tidyverse (version 2.0.0) for data manipulation and visualization, magrittr (version 2.0.3) for piping operations, data.table (version 1.14.8) for high-performance data manipulation, purrr (version 1.0.1) for functional programming, scales (version 1.2.1) for formatting visualizations, psych (version 2.4.3) for summary statistics, broom (version 1.0.4) for regression analysis, car (version 3.1-3) for diagnostic testing, moments (version 0.14.1) for skewness and kurtosis metrics, and effsize (version 0.8.1) for effect size calculations. For text analysis, commercial machine learning models from OpenAI, such as GPT-4-turbo and GPT-3.5 Turbo, were utilized via the Python package openai (version 1.54.4) and the R package rgpt3 (version 0.4). Additionally, R package tweetbotornot2 (version 0.0.1) was used for bot detection on Twitter accounts.

For manuscripts utilizing custom algorithms or software that are central to the research but not yet described in published literature, software must be made available to editors and reviewers. We strongly encourage code deposition in a community repository (e.g. GitHub). See the Nature Portfolio guidelines for submitting code & software for further information.

Data

Policy information about availability of data

All manuscripts must include a data availability statement. This statement should provide the following information, where applicable:

- Accession codes, unique identifiers, or web links for publicly available datasets
- A description of any restrictions on data availability
- For clinical datasets or third party data, please ensure that the statement adheres to our policy

The datasets generated and/or analyzed during the current study are available at the following Zenodo repository (DOI 10.5281/zenodo.15115397): https://zenodo.org/records/15115397.

Due to Twitter's data-sharing policies, raw Twitter data cannot be publicly shared. However, aggregate measures derived from the data are provided. Raw tweets are securely stored on Google's BigQuery SQL database and can be accessed upon reasonable request to the authors, subject to Twitter's policies. Data about academics from OpenAlex is publicly available and can be accessed using the OpenAlex API (https://docs.openalex.org/) or the R package openalexR (version 0.1.1).

We also utilized several publicly available datasets:
- SemEval-2016 Stance Dataset: Available at https://www.saifmohammad.com/WebPages/StanceDataset.htm.
- Gender Classification Dataset: Compiled from the US Social Security Card Applications (1880–2019), UK Baby Names (2011–2018), British Columbia Baby Names (1918–2018), and Australian Baby Names (1944–2019), available from the UC Irvine Machine Learning Repository (https://archive.ics.uci.edu/dataset/591/gender+by+name).
- General Social Survey (GSS): Data available at https://gss.norc.org/us/en/gss/get-the-data.html.
- Random Sample of U.S. Twitter Users: Created by Siegel et al. (2021), with user IDs kindly provided by Pablo Barberá upon request. The tweets and metadata were hydrated using the Twitter API.
- Times Higher Education World University Rankings (2016–2024): Available on Kaggle at https://www.kaggle.com/datasets/raymondtoo/the-world-university-rankings-2016-2024.

This study provides a replication package, including anonymized data and analysis code, ensuring transparency and reproducibility. See Zenodo repository for details.

Research involving human participants, their data, or biological material

Policy information about studies with human participants or human data. See also policy information about sex, gender (identity/presentation), and sexual orientation and race, ethnicity and racism.

Reporting on sex and gender	In this study, the terms sex (biological attribute) and gender (shaped by social and cultural circumstances) were used carefully to avoid confusion. Gender was determined based on self-reporting via OpenAlex profiles and through the use of a large language model (OpenAI's GPT-3.5 Turbo) for name-based classification. The method is described clearly in "Methods" section in subsection 4.6, titled "Gender" and validated in the Appendix. The findings apply to both male and female genders, with 60% of the balanced sample identified as male and 40% as female. No data was collected for non-binary or other gender identities, and consent for sharing individual-level data was not applicable as we used publicly available information. The results show notable differences in political expression across genders, which are detailed in the paper.
Reporting on race, ethnicity, or other socially relevant groupings	Race, ethnicity, and other socially relevant categorization variables were not explicitly collected or analyzed in this study. The focus was on publicly available data regarding academic affiliations, social media activity, and publication records. The lack of race and ethnicity data was due to the constraints of the datasets used (Twitter and OpenAlex), which do not provide this information. The study did, however, examine variations in political expression across different countries, fields of study, and institutional affiliations.
Population characteristics	The study analyzes a sample of 99,274 academics from 12,675 institutions across 174 countries, covering diverse fields of study, including STEM (29.1%), Social Sciences (9.8%), and Humanities (0.5%). The sample includes academics active on Twitter from January 2016 to December 2022, ensuring a balanced representation with activity recorded in both early (January–June 2016) and late (July–December 2022) periods.<br><br>While age data was not available, academic seniority was inferred from publication metrics, such as the number of citations (mean = 1,370.02, SD = 4,523.99), the 2-year impact factor (mean = 16.93, SD = 38.56), and the number of published works (mean = 57.58, SD = 114.38). These proxies provide indirect insights into the career stages of academics in the sample.<br><br>Social media activity levels varied widely: academics averaged 1,575 posts (SD = 2,052.13), 3,211 likes (SD = 5,736.33), 843 retweets (SD = 2,117.91), and 1,113 followers (SD = 9,145.94). Behavioral expression metrics include Egocentrism (mean = 0.33 words per tweet, SD = 0.27), Toxicity (mean = 0.04, SD = 0.04), and Emotionality/Reasoning (mean = 0.75, SD = 0.29).<br><br>The sample exhibited gender diversity, with 60% male and 40% female representation. However, data on non-binary or other gender identities was unavailable due to dataset constraints. Geographically, the dataset includes academics from six continents, with North America and Europe more prominently represented. Comparisons with the general U.S. Twitter population (61,259 users) were conducted to contextualize the findings.<br><br>Key political and behavioral metrics include stances on Climate Action (mean = 0.09, SD = 0.18), Cultural Liberalism (mean = 0.04, SD = 0.08), and Economic Collectivism (mean = 0.01, SD = 0.06). Among 26,555 politically active academics, 75% made at least one non-neutral tweet, with 37%, 56%, and 28% engaging on climate, cultural, and economic topics, respectively.

Recruitment	Participants were included based on their presence in the OpenAlex database and their public Twitter activity. The sample was selected using the high-precision, moderate-recall matching of OpenAlex identifiers to Twitter user IDs (originallly done by Mongeon et al. [2023]), and their data was collected through Twitter's academic API in January 2023. This method ensured a comprehensive and representative sample of academics who engage in social media discourse. Potential biases include self-selection bias, as only academics who actively use Twitter are included, and the use of English-only tweets for certain analyses.
Ethics oversight	Under the Science Engineering Technology Research Ethics Committee (SETREC) process of Imperial College London, the study that has been reviewed by the Research Governance and Integrity Team and Head of Division/Department, where no significant ethical issues have been identified in the protocol or ethics application, can be given RGIT approval without requiring it to go to full committee. SETREC number: 7296355.

Note that full information on the approval of the study protocol must also be provided in the manuscript.

Field-specific reporting

Please select the one below that is the best fit for your research. If you are not sure, read the appropriate sections before making your selection.

☐ Life sciences ☒ Behavioural & social sciences ☐ Ecological, evolutionary & environmental sciences

For a reference copy of the document with all sections, see nature.com/documents/nr-reporting-summary-flat.pdf

Behavioural & social sciences study design

All studies must disclose on these points even when the disclosure is negative.

Study description	This study is a quantitative analysis of social media behavior, focusing on the political and behavioral expression of academics on Twitter. The study uses a novel dataset linking the social media content of around 100,000 academics to their academic records. The data includes various forms of tweets (original posts, retweets, quoted retweets, and replies) and covers a range of politically salient topics such as climate action, cultural, and economic concepts. The analysis employs large-language model-based classification techniques to categorize and label the content, assessing both the substantive content and the tone of the expression.
Research sample	The research sample consists of 99,274 academics from 12,675 institutions across 174 countries and 19 disciplines, as well as a random sample of 61,259 users representative of US Twitter population for comparison. The sample of academics was constructed using a dataset provided by Mongeon et al. (2023), which matched researchers' OpenAlex identifiers with their Twitter/X user identifiers. This comprehensive dataset captures the full Twitter activity of the selected academics from 2016 to 2022, ensuring a balanced sample.
Sampling strategy	The sampling procedure involved selecting academics who had tweeted at least once in the first six months of the sample period (January to June 2016) and the last six months (July to December 2022). This balanced sample includes those actively engaging on Twitter throughout the period. The statistical methods employed included scalable large-language model-based classification techniques to categorize and label the socially shared content. For the general US Twitter population, users were similarly included if they had tweeted at least once in the beginning and end of the sample period, ensuring a balanced comparison. These general users were randomly selected from US Twitter, and originally compiled by Siegel et al. (2021).
Data collection	Data was collected using the Twitter API (academic access) in January 2023. The data includes the full timelines of academics, encompassing original posts, retweets, quoted retweets, and replies. The Twitter data was linked to academic records from OpenAlex, providing comprehensive metadata about the academics' publications, affiliations, and research outputs. Additional data about general US Twitter users was also collected in January 2023 to measure the representation gap in political expression. The raw tweets are securely stored on Google's BigQuery SQL database. Since this study does not involve a randomized controlled trial, the researchers were not blinded to the study hypothesis or conditions
Timing	The data was accessed and collected during the third week of January 2023. The collection lasted around one week due to rate limits in Twitter API.
Data exclusions	Data exclusions involved removing bots from the general US Twitter user sample using the tweetbotornot2 R package. Bots were identified based on a probability threshold, with approximately 5% of users excluded if their bot probability was greater than 50%. Additionally, to ensure a balanced comparison, only users who had tweeted at least once in both the first six months (January to June 2016) and the last six months (July to December 2022) of the sample period were included in the analysis. This balancing criterion was applied to both the academic and general US Twitter user samples.
Non-participation	Since the data was collected from publicly available Twitter accounts and academic records, there were no instances of non-participation or dropouts in the traditional sense. All included users had publicly available data and were active on Twitter during the specified period.
Randomization	Participants were not allocated into experimental groups. The study is observational, focusing on the naturally occurring behavior of academics and general Twitter users.

Reporting for specific materials, systems and methods

We require information from authors about some types of materials, experimental systems and methods used in many studies. Here, indicate whether each material, system or method listed is relevant to your study. If you are not sure if a list item applies to your research, read the appropriate section before selecting a response.

Materials & experimental systems

n/a	Involved in the study
☒	☐ Antibodies
☒	☐ Eukaryotic cell lines
☒	☐ Palaeontology and archaeology
☒	☐ Animals and other organisms
☒	☐ Clinical data
☒	☐ Dual use research of concern
☒	☐ Plants

Methods

n/a	Involved in the study
☒	☐ ChIP-seq
☒	☐ Flow cytometry
☒	☐ MRI-based neuroimaging

Plants

Seed stocks	*Report on the source of all seed stocks or other plant material used. If applicable, state the seed stock centre and catalogue number. If plant specimens were collected from the field, describe the collection location, date and sampling procedures.*
Novel plant genotypes	*Describe the methods by which all novel plant genotypes were produced. This includes those generated by transgenic approaches, gene editing, chemical/radiation-based mutagenesis and hybridization. For transgenic lines, describe the transformation method, the number of independent lines analyzed and the generation upon which experiments were performed. For gene-edited lines, describe the editor used, the endogenous sequence targeted for editing, the targeting guide RNA sequence (if applicable) and how the editor was applied.*
Authentication	*Describe any authentication procedures for each seed stock used or novel genotype generated. Describe any experiments used to assess the effect of a mutation and, where applicable, how potential secondary effects (e.g. second site T-DNA insertions, mosiacism, off-target gene editing) were examined.*
